# Six polymorphisms in the lncRNA H19 gene and the risk of cancer: a systematic review and meta-analysis

**DOI:** 10.1186/s12885-023-11164-y

**Published:** 2023-07-21

**Authors:** Maoquan Yang, Mingwei Zhang, Qiong Wang, Xiaojing Guo, Peizhen Geng, Jinhua Gu, Wansheng Ji, Li Zhang

**Affiliations:** 1 School of Clinical Medicine, Affiliated Hospital of Weifang Medical University, Weifang Medical University, Weifang, Shandong China; 2 Department of Gastroenterology, Affiliated Hospital of Weifang Medical University, No. 2428, Yuhe Road, Weifang, 261031 Shandong China; 3 Department of Pathology, Shandong University School of Basic Medical Sciences, Jinan, Shandong China; 4 Department of Gastroenterology, Qilu Hospital of Shandong University, Jinan, Shandong China; 5 Department of Gastroenterology, Weifang NO.2 People s Hospital, Weifang, Shandong China; 6 Experimental Center for Medical Research, Weifang Medical University, Weifang, Shandong China

**Keywords:** Cancer, H19, Gene polymorphism, Cancer, Susceptibility, Meta-analysis

## Abstract

**Background:**

Numerous studies have demonstrated long noncoding RNA (lncRNA) play an important role in the occurrence and progression of cancer, and single nucleotide polymorphisms (SNPs) located in lncRNA are considered to affect cancer suspensibility. Herein, a meta-analysis was carried out to better assess the relationship of H19 polymorphisms and cancer susceptibility.

**Methods:**

A literature search was conducted through using PubMed, EMBASE, and Web of Science databases to obtain relevant publications before Aug 23, 2022. The reference lists of the retrieved studies were also investigated to identify additional relevant articles. The pooled odds ratios (ORs) with 95% confidence intervals (CIs) were calculated to appraise the risk of various cancers.

**Results:**

There appeared to be a remarkable correlation between the rs2107425 variation and decreased cancer risk among Caucasians. Nevertheless, the rs217727 polymorphism was significantly associated with an increased risk of lung cancer, hepatocellular carcinoma and oral squamous cell carcinoma. Also, we found a significant correlation between the rs2839698 polymorphism and increased cancer risk among Asians, gastric cancer, hepatocellular carcinoma, hospital-based control and larger simple size subgroups, respectively. Similarly, the rs3741219 mutation was notably related to cancer risk in higher quality score. As for rs3024270 polymorphism, the homozygous model was markedly linked to cancer risk in overall analysis and population-based controls. There was no significant association between the rs3741216 polymorphism and cancer risk.

**Conclusion:**

H19 rs2839698 and rs3024270 were closely associated with overall cancer risk. H19 rs2107425 was related to lower cancer risk among Caucasians, while the rs2839698 was related to increased cancer risk among Asians. Our results supported that H19 SNPs were significantly correlated with cancer risk.

**Supplementary Information:**

The online version contains supplementary material available at 10.1186/s12885-023-11164-y.

## Introduction

Cancer has been the second biggest cause of mortality worldwide, seriously endangering public health and increasing economic burden on society [1]. In 2023, 1,918,030 new cancer cases and 609,360 cancer mortalities are estimated to occur in the United States. And prostate, lung, and colorectal cancers account for 48% of all male incident cases, while 51% of all female incident cases are diagnosed with breast, lung, and colorectal cancers [2]. Although the specific pathological mechanism of tumorigenesis still remains unclear, cancer is considered as a complex and multifactorial disease that results from the interaction of environmental and genetic risk factors, such as high-calorie diet, smoking, excessive drinking, obesity, hypertension, diabetes [3–5]. Recent advances in cancer diagnosis and treatment, including multifunctional nanomaterials combined with imaging probes and drugs, nanomedicine products and therapeutic vaccines are improving options for cancer patients [6–8]. On the other hand, preference heterogeneity between patients indicates that tradeoffs between survival benefits and long-term physical, emotional, cognitive, and functional side effects should be carefully considered in treatment decision-making [9]. At present, genome-wide association studies (GWAS) have identified a strong association of several common single nucleotide polymorphisms (SNPs) with cancer risk [10, 11]. Certain genetic SNPs were found to be related to cancer risk, including miR-143/145, CASP9, CASP10 and IL-1β [12–14]. In addition, functional SNPs are present in lncRNA genes and influence gene expression and function through various means, and then result in the occurrence and progression of cancer [15].

Being widely transcribed in the human genome, long non-coding RNAs (lncRNAs) are defined as single stranded non-coding RNAs with a length of more than 200 base pairs and no open reading frames, thereby lacking of protein-coding function, although some of them may produce small functional peptides [16]. LncRNAs take part in numerous cellular processes by interacting with cellular molecules, such as DNAs, RNAs, or proteins [17]. At the levels of epigenetic, transcriptional, and post-transcriptional modifications, they can regulate gene expression via different mechanisms, including chromatin remodeling induction, alternative splicing, intranuclear transport, production of miRNA sponges, and transcriptional interference [18–22]. Interestingly, lncRNAs paly crucial regulatory roles in a variety of physiological and pathological processes and cancer biology, including cell proliferation, differentiation, apoptosis, and carcinogenesis progression [23–27]. It has been found that lncRNAs are dysregulated in various types of cancer, which contributes to tumorigenesis and development of tumors by affecting the expression of oncogenes or tumor suppressors. Generally, lncRNAs are thought to have prospective clinical implications and to be appraised as independent novel biomarkers for diagnosis and prognosis in human cancer treatment [28–30].

As a critical maternally imprinted gene, lncRNA H19 was initially discovered in the 1990s [31]. The H19 gene, possessing five exons and four introns, encodes a 2.3-kb long, capped, spliced, and polyadenylated noncoding RNA, of which the transcript is highly conserved at a cluster with the insulin-like growth factor 2 (IGF2) locus on human chromosome 11p15.5, and plays an essential role in embryonic development and growth control [32–35]. It has been reported that the aberrant expression of H19 was implicated in various types of cancer, including breast, lung, esophageal, gastric, pancreatic, colorectal, liver, bladder and cervical cancer. H19 acts as an oncogene or a suppressor gene, which may be attributed to the heterogeneity of different types of cancer [36–38]. Previous researches have shown that H19 gene polymorphisms are markedly associated with malignancies, however, the results were controversial and inconsistent. Therefore, the aim of this meta-analysis was to accurately examine the correlation between H19 polymorphisms and cancer susceptibility.

## Materials and methods

### Literature search strategy

Eligible studies were retrieved from the PubMed, EMBASE, and Web of Science electronic databases up to Aug 23, 2022. Our search strategy included the main terms for: (H19 or long Noncoding RNA H19 or lncRNA H19) and (polymorphism or genotype or SNP) and (carcinoma or neoplasm or cancer or tumor). At the same time, we manually screened out the relevant potential articles in the references extracted.

### Selection and exclusion criteria

Inclusion criteria are as follows: (1) case-control studies investigated the relationship between H19 polymorphisms and the risk of cancer; (2) the histopathological diagnosis of cancer patients was clearly defined; the control group did not have any history of cancer; (3) sufficient data on genotype distribution of H19 polymorphisms was applied to calculate the odds ratio (OR) and 95% confidence interval (CI).

The exclusion criteria were as follows: (1) abstract, case reports, comment, editorials and review; (2) duplication of the previous reports; (3) lack of the full text or main genotyping data; (4) non-case-control or cohort design studies.

### Data extraction

Two investigators separately conducted literature screening, data extraction, literature quality evaluation, and any disagreements that could be resolved through discussion or a third analyst. The relevant information independently extracted by two investigators included the following information from each study: first author, year of publication, country of the population, ethnicity, source of controls, genotyping methods, cancer types, sample size and P value of (HWE).

The Newcastle-Ottawa scale (NOS) was adopted to assess the process in terms of queue selection, comparability of queues, and evaluation of results [39]. A study with a score of at least six was considered as a high-quality literature. Higher NOS scores showed higher literature quality.

### Statistical analysis

All data analysis was conducted using Stata16.0 software (Stata Corp LP, TX, USA). Odds ratio (OR) and 95% confidence intervals (CIs) were used to evaluate the association between lncRNA H19 polymorphisms and various cancers. After that, the heterogeneity test was conducted. When P ≥ 0.05 or I^2^ < 50% was performed, it indicated that there was no obvious heterogeneity, and the fixed-effect pattern should be applied for a merger. Otherwise, the random-effect model was used. Results were considered significant statistically when the p-value less than 0.05. Subgroup analysis was implemented to determine the source of heterogeneity. Additionally, sensitivity analysis was performed to assess the impact of each individual study on overall results. The Begg’s rank correlation test and Egger’s linear regression test were used to verify the publication bias among these studies. If P < 0.05 indicates obvious publication bias.

### False-positive report probability (FPRP) analysis

The probability of meaningful relationships between H19 SNPs and cancer risk can be determined through carrying out the FPRP analysis [40]. In order to investigate the remarkable associations observed in the meta-analysis, we adopted prior probabilities of 0.25, 0.1, 0.01, 0.001, and 0.0001 and computed the FPRP values as described previously. The association that reached the FPRP threshold of < 0.2 was considered significant.

## Results

### Process of study selection and description of qualified studies

**Fig. 1:**
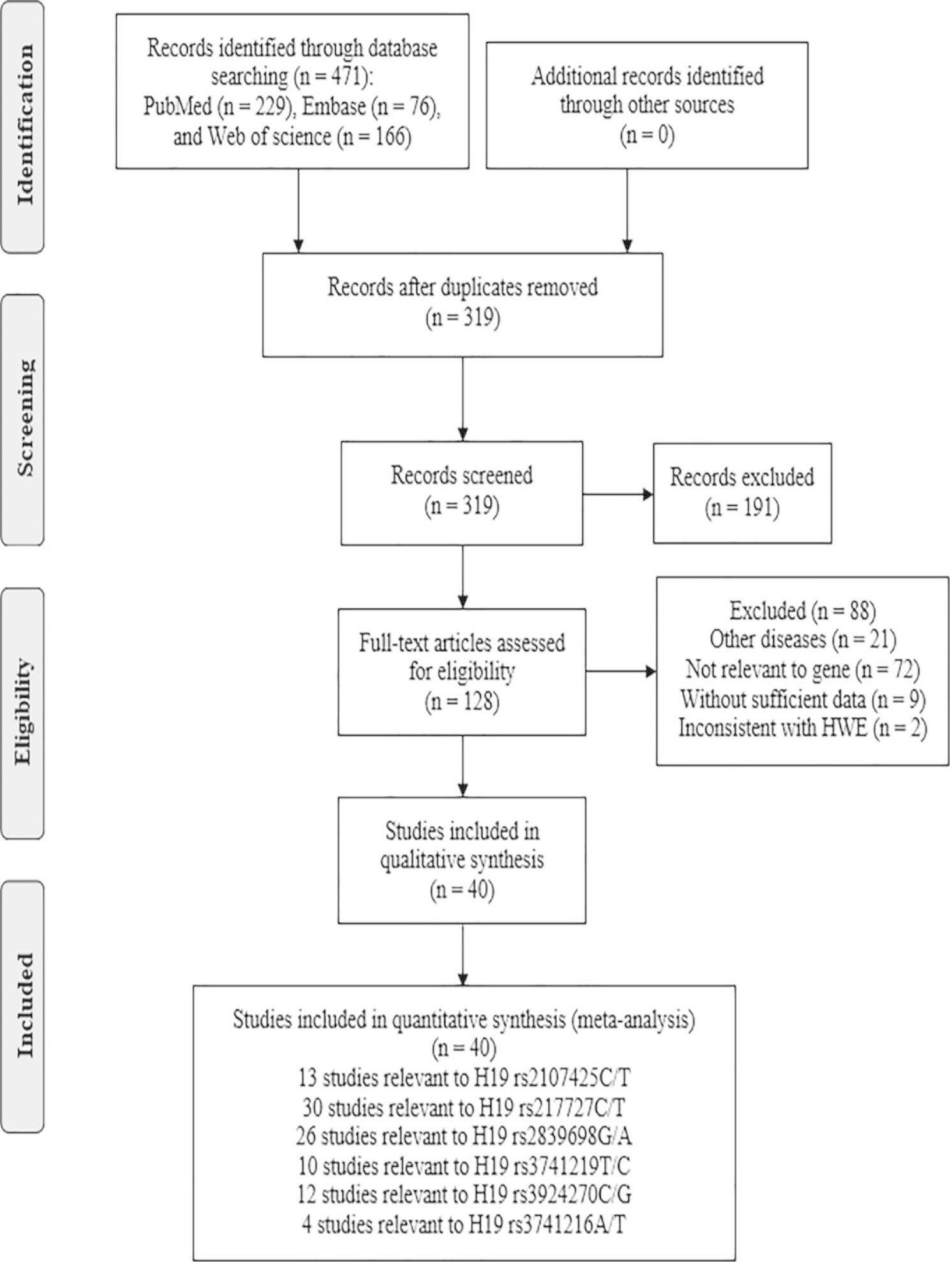
Flow diagram of the eligible study selection process

As shown in Fig. 1, **t**he initial 472 studies were retrieved by databases of PubMed (n = 229), Embase (n = 76), Web of science (n = 166). After eliminating 152 duplicate articles, 191 additional publications were excluded by screening the abstract and title. Among these, 147 articles were reviews, letters, conference abstracts, meta-analysis, notes, editorials and short surveys, and 44 articles focused on animal or vitro experiment. After careful review of the full texts, 88 articles were further excluded due to the following reasons: 30 articles were involved with other genes or other SNPs of H19, 45 studies were not relevant to cancer and 13 studies had no available data. Finally, the remaining 40 eligible articles were included in this analysis [41–80].

Through literature search and selection, a total of 40 eligible articles embodying 95 studies were embodying in our study, which included 13 studies for rs2107425, 30 studies for rs217727, 26 studies for rs2839698, 10 studies for rs3741219, 12 studies for rs3024270, and 4 studies for rs3741216 polymorphisms. One article referred to two independent case-control studies, and thus the study was regarded as two separate estimates [44]. Among the included studies, 30 studies were from China, four studies from Iran, two studies form European countries, two studies from Egypt, two studies from the mixed countries, and one study from America. At the same time, 34 studies were conducted in the Asian descent, five studies were conducted in the Caucasian descent and two studies were conducted in the African descent. Thirteen of the studies focused on population-based controls and 27 on hospital-based controls. If the number of different cancer types is less than 1, the cancer type is classified into other cancer subgroup. The detailed characteristics of selected studies are illustrated in Table 1, such as cancer type, genotyping method, sample size, distributions of genotype frequency and Hardy-Weinberg equilibrium. The NOS score of all articles ranged from 6 to 8, implying that all included studies were of high quality.

**Table 1:** Characteristics of all studies included in the meta-analysis

First author	Year	Country	Ethnicity	Genotyping	Source of	Genotype distribution						HWE	NOS	Cancer
				methods	control	Case			Control			P-value		types
**13 Studies for H19 gene rs2107425 C/T polymorphism**														
						CC	CT	TT	CC	CT	TT			
Verhaegh	2008	Netherlands	Caucasian	PCR-RFLP	PB	92	65	20	89	96	19	0.3402	9	BLC
Song HL	2009	Mixed	Caucasian	TaqMan	PB	2619	2192	555	4029	3667	842	0.8565	9	OC
Quaye	2009	Mixed	Caucasian	TaqMan	PB	767	544	149	1118	1098	247	0.3449	6	OC
Barnholtz	2010	USA	African	Illumina	PB	161	390	186	170	339	149	0.4199	7	BC
Barnholtz	2010	USA	Caucasian	Illumina	PB	604	516	105	521	478	119	0.5489	7	BC
Butt S	2012	Sweden	Caucasian	Mass Array	PB	361	250	68	668	573	145	0.1816	8	BC
Gong WJ	2016	China	Asian	Sequenom	HB	181	235	63	79	96	28	0.8920	6	LC
Yin ZH	2018	China	Asian	Illumina	HB	161	266	129	140	185	70	0.5129	8	LC
Wu	2019	China	Asian	TaqMan	HB	134	185	40	422	560	208	0.3451	8	HCC
Huang MC	2019	China	Asian	PCR	PB	88	107	38	109	155	48	0.5590	8	CC
Yang PJ	2019	China	Asian	RT-PCR	HB	152	213	66	171	190	70	0.1636	7	UCC
Ghapanchi	2020	Iran	Asian	ARMS-PCR	PB	79	94	27	74	101	25	0.2911	8	OSCC
Khalil	2022	Egypt	African	QIAamp	HB	25	32	13	20	9	1	0.9919	6	CRC
**30 Studies for H19 gene rs217727 G/A polymorphism**														
						GG	GA	AA	GG	GA	AA			
Verhaegh	2008	Netherlands	Caucasian	PCR-RFLP	PB	114	59	4	115	80	9	0.2880	9	BLC
Yang C	2015	Netherlands	Caucasian	TaqMan	HB	160	252	88	193	244	63	0.2957	8	GC
Li SW	2016	China	Asian	TaqMan	HB	480	514	153	456	570	177	0.9585	9	CRC
Hua QH	2016	China	Asian	TaqMan	HB	431	467	148	573	665	156	0.0740	7	BLC
Xia Z	2016	China	Asian	CRS-RFLP	PB	160	156	148	139	212	116	0.0521	9	BC
Jin TB	2016	China	Asian	Mass Array	PB	117	103	26	169	99	16	0.7651	9	CC
Guo QY	2017	China	Asian	Illumina	HB	101	181	80	252	348	137	0.3840	8	OSCC
Hassanzarei	2017	China	Asian	PCR-RFLP	HB	71	132	27	125	113	2	0.0000^*^	7	BC
He TD	2017	Iran	Asian	TaqMan	HB	79	102	12	195	165	23	0.1207	6	Osteosarcoma
Hu PH	2017	China	Asian	TaqMan	HB	133	200	83	128	196	92	0.3022	8	PC
Lin YX	2017	China	Asian	G0104K	HB	403	471	131	465	450	105	0.8007	8	BC
Li LL	2018	China	Asian	TaqMan	HB	210	250	95	246	305	67	0.0542	8	LC
Yin ZH	2018	China	Asian	Illumina	HB	204	264	88	165	172	58	0.2319	8	LC
Yuan ZY	2018	China	Asian	Mass Array	PB	186	194	51	488	423	73	0.1511	7	OSCC
Cui P	2018	China	Asian	TaqMan	PB	611	692	185	685	773	217	0.9628	7	BC
						GG	GA	AA	GG	GA	AA			
Abdollahzadeh	2018	China	Asian	RFLP-PCR	HB	116	29	5	86	14	0	0.4516	8	BC
Hu C	2019	Iran	Asian	TaqMan	HB	186	164	43	382	342	86	0.4696	7	Neuroblastoma
Li Z	2019	China	Asian	TaqMan	HB	51	140	9	84	90	26	0.8061	8	BLC
Mohammad	2019	China	Asian	ARMS-PCR	HB	79	30	2	64	54	12	0.9003	7	BC
Wang GZ	2019	Iran	Asian	TaqMan	HB	162	277	125	493	751	291	0.8676	6	LC
Wu	2019	China	Asian	TaqMan	HB	154	170	35	495	539	156	0.6265	8	HCC
Wei MR	2019	China	Asian	TaqMan	HB	88	72	65	63	44	93	0.0000^*^	7	GC
Huang MC	2019	China	Asian	PCR	PB	102	103	28	135	139	39	0.7289	8	CC
Yang PJ	2019	China	Asian	RT-PCR	HB	185	202	44	191	188	52	0.5845	7	UCC
Cao Q	2020	China	Asian	RT-PCR	HB	343	550	201	350	494	183	0.7042	8	RCC
Ghapanchi	2020	China	Asian	ARMS-PCR	PB	110	75	15	133	64	3	0.1259	8	OSCC
Deng YJ	2020	Iran	Asian	Mass Array	HB	254	278	73	557	591	152	0.8018	7	Glioma
Tan TB	2020	China	Asian	TaqMan	HB	126	68	19	438	410	109	0.3811	8	Hepatoblastoma
Li WY	2021	China	Asian	TaqMan	PB	177	130	48	486	469	113	0.9925	7	Wilms
Pei JS	2021	China	Asian	RT-PCR	PB	111	120	35	114	120	32	0.9610	7	Leukemia
**26 Studies for H19 gene rs2839698 G/A polymorphism**														
						GG	GA	AA	GG	GA	AA			
Verhaegh	2008	Netherlands	Caucasian	PCR-RFLP	PB	54	74	49	52	109	43	0.3125	9	BLC
Yang C	2015	China	Asian	TaqMan	HB	250	195	55	284	178	38	0.1754	8	GC
Li SW	2016	China	Asian	TaqMan	HB	583	462	102	666	462	75	0.6665	9	CRC
Hua QH	2016	China	Asian	TaqMan	HB	552	418	79	729	565	103	0.6510	7	BLC
Gong WJ	2016	China	Asian	TaqMan	HB	237	220	39	99	80	27	0.0982	6	LC
Guo QY	2017	China	Asian	Illumina	HB	133	171	58	244	377	120	0.2021	8	ORC
Hassanzarei	2017	China	Asian	PCR-RFLP	HB	0	64	166	0	18	222	0.5461	7	BC
He TD	2017	Iran	Asian	TaqMan	HB	83	98	12	178	175	30	0.1462	6	Osteosarcoma
Lin YX	2017	China	Asian	G0104K	HB	452	440	113	484	432	104	0.5998	8	BC
Yang ML	2018	China	Asian	KASP	HB	215	211	40	245	185	32	0.7141	8	HCC
Cui P	2018	China	Asian	TaqMan	PB	801	568	122	875	673	129	0.9793	7	BC
Hu C	2019	China	Asian	TaqMan	HB	179	175	39	365	373	72	0.0896	7	Neuroblastoma
Mohammad	2019	China	Asian	ARMS-PCR	HB	15	57	39	53	55	22	0.2410	7	BC
Wang GZ	2019	China	Asian	TaqMan	HB	277	225	61	712	645	175	0.1173	6	LC
Wu	2019	China	Asian	TaqMan	HB	140	178	41	532	524	134	0.7718	8	HCC
Wei MR	2019	Iran	Asian	TaqMan	HB	90	68	67	88	78	34	0.0248^*^	7	GC
Huang MC	2019	China	Asian	PCR	PB	115	99	20	154	134	30	0.9132	8	CC
						GG	GA	AA	GG	GA	AA			
Yang PJ	2019	China	Asian	RT-PCR	HB	206	170	55	192	184	55	0.2973	7	UCC
Cao Q	2020	Iran	Asian	RT-PCR	HB	516	435	76	615	425	54	0.0732	8	RCC
Deng YJ	2020	China	Asian	PCR	HB	134	140	40	154	211	72	0.0581	7	Glioma
Yu BQ	2020	China	Asian	Mass Array	HB	311	240	54	675	504	121	0.9847	7	CRC
Zhang HB	2020	China	Asian	Mass Array	HB	70	93	38	92	88	16	0.4260	6	OC
Tan TB	2020	China	Asian	TaqMan	PB	102	78	33	439	424	94	0.5679	8	Hepatoblastoma
Li WY	2021	China	Asian	TaqMan	HB	174	127	54	488	480	100	0.2453	7	Wilms
Pei JS	2021	China	Asian	RT-PCR	PB	91	131	44	119	117	30	0.8781	7	Leukemia
Zhang JZ	2021	Iran	Asian	PCR-RFLP	HB	192	244	137	351	248	89	0.0000^*^	8	Lymphoma
**10 Studies for H19 gene rs3741219 A/G polymorphism**														
						AA	AG	GG	AA	AG	GG			
Yang C	2015	China	Asian	TaqMan	HB	260	187	53	268	189	43	0.2446	8	GC
Hassanzarei	2017	Iran	Asian	PCR-RFLP	HB	63	126	42	109	102	29	0.4979	7	BC
Cui P	2018	China	Asian	TaqMan	PB	782	582	127	832	706	139	0.5291	7	BC
Abdollahzadeh	2018	Iran	Asian	RFLP-PCR	HB	119	24	7	80	17	3	0.0993	8	BC
Wu	2019	China	Asian	TaqMan	HB	129	182	48	517	536	137	0.9140	8	HCC
Yang PJ	2019	China	Asian	RT-PCR	HB	192	181	58	185	190	56	0.5121	7	UCC
Huang MC	2019	China	Asian	PCR	PB	112	100	21	152	130	32	0.5906	8	CC
Cao Q	2020	China	Asian	RT-PCR	HB	567	416	111	552	389	86	0.1400	8	RCC
Deng YJ	2020	China	Asian	Mass Array	HB	439	107	59	651	520	129	0.0956	7	Glioma
Zhang HB	2020	China	Asian	Mass Array	HB	190	1	20	186	0	9	0.0000^*^	6	OC
**12 Studies for H19 gene rs3024270 C/G polymorphism**														
						CC	CG	GG	CC	CG	GG			
Hua QH	2016	China	Asian	TaqMan	HB	174	527	346	260	688	447	0.8686	7	BLC
Li SW	2016	China	Asian	TaqMan	HB	385	527	235	420	582	201	0.9794	9	CRC
Guo QY	2017	China	Asian	Illumina	HB	75	183	104	145	350	245	0.3213	8	OSCC
He TD	2017	China	Asian	TaqMan	HB	17	91	85	31	179	173	0.1014	6	Osteosarcoma
Yang ML	2018	China	Asian	KASP	HB	95	225	151	81	215	170	0.3609	8	HCC
Hu C	2019	China	Asian	TaqMan	HB	99	203	91	213	424	173	0.1591	7	Neuroblastoma
Li Z	2019	China	Asian	TaqMan	HB	16	101	83	22	97	81	0.3771	8	BLC
Huang MC	2019	China	Asian	PCR	PB	51	120	60	71	150	95	0.4225	8	CC
Wu	2019	China	Asian	TaqMan	HB	87	187	85	334	593	263	0.9945	8	HCC
Yang PJ	2019	China	Asian	RT-PCR	HB	114	210	107	120	208	103	0.4894	7	UCC
Tan TB	2020	China	Asian	TaqMan	PB	50	87	76	264	489	204	0.4216	8	Hepatoblastoma
						CC	CG	GG	CC	CG	GG			
Li WY	2021	China	Asian	TaqMan	HB	120	141	94	290	556	222	0.1376	7	Wilms
**4 Studies for H19 gene rs3741216 A/T polymorphism**														
						AA	AT	TT	AA	AT	TT			
Yang C	2015	China	Asian	TaqMan	HB	380	102	18	379	109	12	0.2210	8	GC
Hassanzarei	2017	Iran	Asian	PCR-RFLP	HB	0	26	204	0	65	26	0.0150^*^	7	BC
Wei MR	2019	China	Asian	TaqMan	HB	79	91	55	70	78	52	0.0025^*^	7	GC
Cao Q	2020	China	Asian	RT-PCR	HB	791	255	48	718	264	35	0.0834	8	RCC

BC: breast cancer; LC: lung cancer; BLC: bladder cancer; GC: gastric cancer; CRC: colorectal cancer; PC: pancreatic cancer; OC: ovarian cancer; CC: cervical cancer; OSCC: oral squamous cell carcinoma; UCC: urothelial cell carcinoma; RCC: renal cell carcinoma. ^*^P < 0.05

### Correlation between rs2107425 C/T polymorphism and cancer risk

**Fig. 2:**
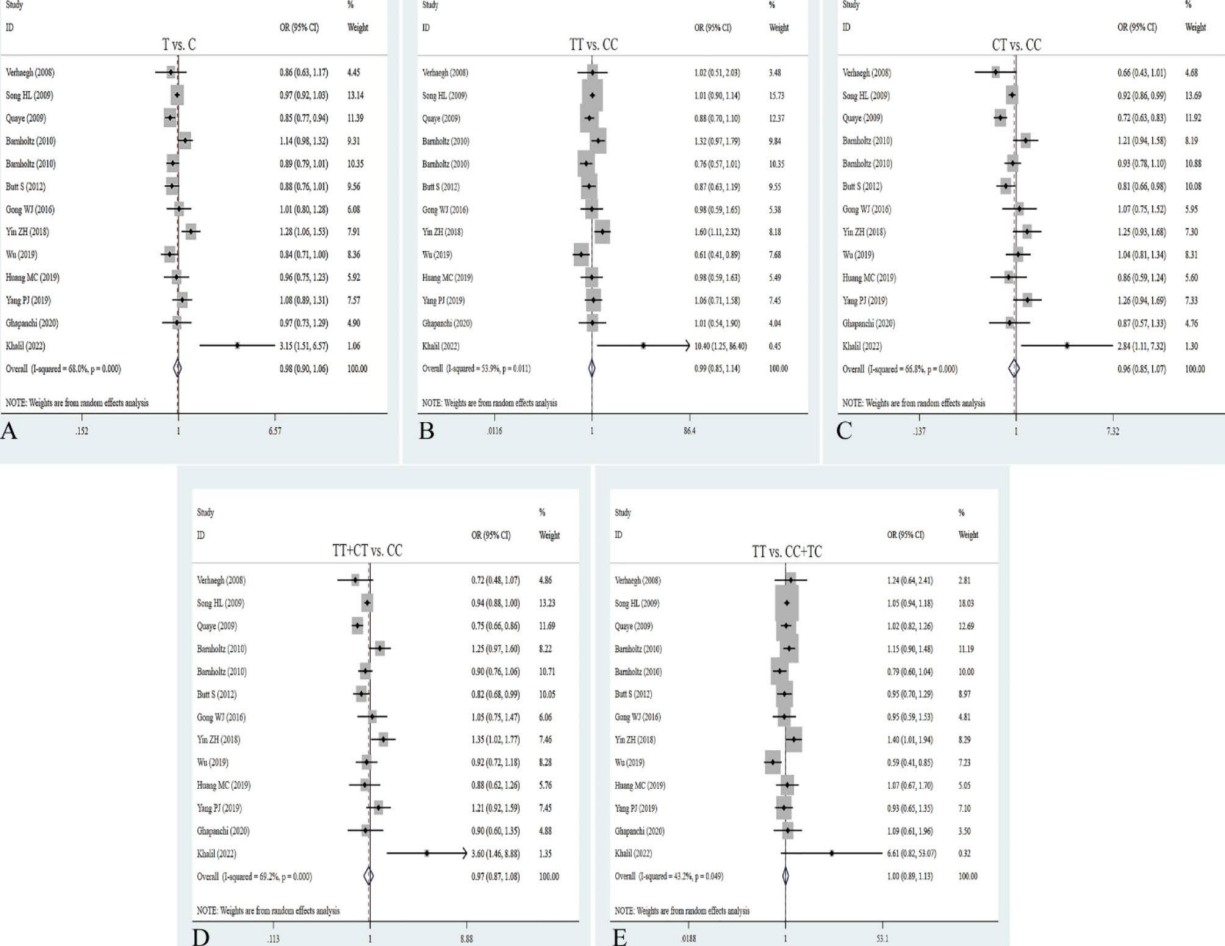
Forest plots for the association between H19 rs2107425 polymorphism and cancer risk in five models. **A**: allele model; **B**: dominant model; **C**: heterozygote model; **D**: homozygote model; **E**: recessive model

Thirteen relevant studies with 11,972 cancer patients and 17,128 controls were examined for the association between the rs2107425 polymorphism and cancer risk. Compared with the wild-type CC homozygote, the genotypes of rs2107425 were not linked to cancer risk in overall analyses (T vs. C: OR = 0.98, 95%CI = 0.91–1.06, *P* = 0.595; TT vs. CC: OR = 1.01, 95%CI = 0.88–1.17, *P* = 0.846; TC vs. CC: OR = 0.96, 95%CI = 0.85–1.07, *P* = 0.438). Similarly, no relationships were detected in the dominant and recessive models (TT + TC vs. CC: OR = 0.97, 95%CI = 0.87–1.08, *P* = 0.543; TT vs. TC + CC: OR = 0.98, 95%CI = 091-1.06, *P* = 0.651; Table 2; Fig. 2). Stratification analysis by ethnicity showed the rs2107425 variation significantly reduced cancer risk among Caucasians (T vs. C: OR = 0.91, 95% CI = 0.85 − 0.7, *P* = 0.006; CT vs. CC: OR = 0.83, 95% CI = 0.73–0.94, *P* = 0.003; OR = 0.85, 95% CI = 0.76–0.94, *P* = 0.003), which might be a protective factor in the Caucasian population. Also, we found a significant association of rs2107425 variant with cancer risk under the heterozygote models in hospital-based subgroup (CT vs. CC: OR = 1.18, 95% CI = 1.00-1.39, *P* = 0.049) and population-based source of controls (CT vs. CC: OR = 0.87, 95% CI = 0.78–0.97, *P* = 0.016, Table 2). There was significant association between the rs2107425 variant and elevated risk of CRC (T vs. C: OR = 3.15, 95%CI = 1.51–6.57, *P* = 0.002; TT vs. CC: OR = 10.40, 95%CI = 0.1.25–86.4, *P* = 0.030; TC vs. CC: OR = 2.84, 95%CI = 1.11–7.32, *P* = 0.030; TT + TC vs. CC: OR = 3.60, 95%CI = 1.46–8.88, *P* = 0.005). The heterozygote and recessive models of rs2107425 notably decreased the risk of hepatocellular carcinoma (TT vs. CC: OR = 0.61, 95%CI = 0.41–0.90, *P* = 0.012; TT vs. CC + TC: OR = 0.59, 95%CI = 0.41–0.85, *P* = 0.004, Table 2). Heterogeneity test results suggested that heterogeneity existed in all five genetic models of overall analyses. Heterogeneity was not observed under the allelic, homozygote, and recessive models in Caucasians subgroup.

**Table 2:** Summary ORs and 95% CIs of H19 SNPs and risk of cancer

Locus	No.	Allele		Homozygote		Heterozygote		Dominant		Recessive	
		OR (95%CI) *P*	I^2^ (%)	OR (95%CI) *P*	I^2^ (%)	OR (95%CI) *P*	I^2^ (%)	OR (95%CI) *P*	I^2^ (%)	OR (95%CI) *P*	I^2^ (%)
rs2107425C/T											
Total	13	0.98 (0.91, 1.06) 0.595	68.0	1.01 (0.88, 1.17) 0.846	53.9	0.96 (0.85, 1.07) 0.438	66.8	0.97 (0.87, 1.08) 0.543	69.2	0.98 (0.91, 1.06) 0.651	43.2
Ethnicity											
Caucasian	5	0.91 (0.85, 0.97) 0.006^*^	46.2	0.94 (0.85, 1.04) 0.226	6.4	0.83 (0.73, 0.94) 0.003^*^	67.0	0.85 (0.76, 0.94) 0.003^*^	62.3	1.01 (0.93, 1.11) 0.813	5.1
Asian	6	1.02 (0.89, 1.16) 0.799	54.9	1.00 (0.75, 1.35) 0.978	60.6	1.08 (0.95, 1.23) 0.249	0.0	1.06 (0.92, 1.22) 0.453	24.6	0.96 (0.82, 1.13) 0.649	60.1
African	2	1.77 (0.66, 4.76) 0.255	85.8	2.82 (0.39, 20.15) 0.302	72.5	1.64 (0.74, 3.63) 0.224	65.5	1.93 (0.69, 5.38) 0.208	79.7	1.21 (0.95, 1.54) 0.132	62.8
Source of control											
PB	8	0.94 (0.87, 1.00) 0.067	50.1	0.97 (0.87, 1.08) 0.529	16.8	0.87 (0.78, 0.97) 0.016^*^	61.6	0.89 (0.80, 0.98) 0.024	60.5	1.03 (0.95, 1.12) 0.519	0.0
HB	5	1.13 (0.90, 1.52) 0.313	79.3	1.11 (0.69, 1.77) 0.664	76.8	1.18 (1.00, 1.39) 0.049^*^	18.4	1.20 (0.94, 1.53) 0.143	63.3	0.96 (0.80, 1.15) 0.475	74.1
NOS scores											
N1	6	1.02 (0.88, 1.20) 0.761	78.9	1.00 (0.79, 1.28) 0.977	59.6	1.06 (0.83, 1.35) 0.658	81.3	1.07 (0.84, 1.35) 0.593	82.3	1.02 (0.90, 1.16) 0.778	7.3
N2	7	0.96 (0.87, 1.06) 0.414	55.6	0.98 (0.80, 1.20) 0.839	55.4	0.93 (0.82, 1.03) 0.129	36.1	0.93 (0.83, 1.04) 0.197	43.0	0.97 (0.89, 1.07) 0.568	62.3
Sample size											
S1	7	1.08 (0.92, 1.26) 0.344	60.1	1.17 (0.91, 1.49) 0.221	28.6	1.04 (0.84, 1.31) 0.708	56.1	1.08 (0.86, 1.36) 0.492	62.4	1.15 (0.96, 1.37) 0.215	3.3
S2	6	0.93 (0.85, 1.06) 0.061	67.5	0.91 (0.76, 1.07) 0.249	63.7	0.91 (0.80, 1.03) 0.115	71.8	0.90 (0.80, 1.01) 0.070	69.7	0.99 (0.91, 1.08) 0.830	62.2
Cancer type											
OC	2	0.92 (0.80, 1.05) 0.196	82.5	0.98 (0.86, 1.11) 0.698	18.4	0.82 (0.65, 1.04) 0.102	89.2	0.85 (0.68, 1.05) 0.127	88.6	1.05 (0.95, 1.16) 0.371	0.0
BC	3	0.96 (0.82, 1.13) 0.624	74.6	0.95 (0.69, 1.32) 0.774	71.6	0.96 (0.78, 1.17) 0.661	66.9	0.96 (0.77, 1.19) 0.683	73.0	0.97 (0.83, 1.13) 0.681	51.3
LC	2	1.15 (0.92, 1.44) 0.226	56.0	1.30 (0.81, 2.09) 0.280	65.1	1.17 (0.94, 1.47) 0.168	0.0	1.21 (0.95, 1.54) 0.119	21.0	1.24 (0.95, 1.63) 0.114	43.7
rs217727G/A											
Total	30	1.06 (0.99, 1.14) 0.097	77.6	1.12 (0.97, 1.30) 0.109	70.2	1.07 (0.97, 1.17) 0.182	70.2	1.08 (0.98, 1.19) 0.109	74.4	1.09 (0.96, 1.24) 0.201	70.1
Ethnicity											
Caucasian	1	0.74 (0.52, 1.05) 0.089		0.49 (0.13, 1.50) 0.192		0.74 (0.49, 1.14) 0.172		0.71 (0.47, 1.08) 0.111		0.50 (0.15, 1.66) 0.257	
Asian	29	1.07 (1.00, 1.15) 0.060	77.6	1.14 (0.98, 1.31) 0.082	70.6	1.08 (0.98, 1.18) 0.130	70.5	1.09 (0.99, 1.20) 0.070	74.5	1.10 (0.96, 1.025) 0.165	70.6
Source of control											
PB	9	1.06 (0.91, 1.24) 0.460	79.2	1.18 (0.87, 1.06) 0.286	70.6	0.96 (0.79, 1.17) 0.689	73.9	1.01 (0.83, 1.24) 0.903	77.4	1.21 (0.94, 1.57) 0.143	63.9.
HB	21	1.07 (0.98, 1.16) 0.144	77.9	1.11 (0.94, 1.31) 0.236	71.5	1.11 (1.00, 1.23) 0.055	68.7	1.11 (1.00, 1.24) 0.063	73.7	1.05 (0.90, 1.23) 0.567	73.0
NOS scores											
N1	11	1.03 (0.89, 1.19) 0.068	84.7	1.12 (0.85, 1.47) 0.441	77.9	1.05 (0.90, 1.22) 0.558	70.7	1.04 (0.88, 1.23) 0.063	78.8	1.10 (0.94, 1.27) 0.529	78.1
N2	19	1.08 (0.99, 1.17) 0.717	71.4	1.13 (0.96, 1.34) 0.145	65.4	1.08 (0.95, 1.22) 0.229	71.3	1.10 (0.98, 1.24) 0.103	72.5	1.09 (0.84, 1.41) 0.240	64.7
Sample size											
S1	14	1.08 (0.90, 1.29) 0.432	84.3	1.11 (0.76, 1.60) 0.595	76.5	1.15 (0.92, 1.44) 0.215	77.9	1.14 (0.91, 1.42) 0.272	81.1	1.03 (0.68, 1.40) 0.873	79.2
S2	16	1.06 (0.99, 1.13) 0.094	67.8	1.17 (1.02, 1.33) 0.022^*^	61.9	1.02 (0.94, 1.11) 0.636	57.7	1.05 (0.96, 1.15) 0.290	65.1	1.14 (1.03, 1.28) 0.015^*^	52.0
Cancer type											
BLC	3	1.01 (0.82, 1.25) 0.923	56.8	0.80 (0.40, 1.62) 0.538	64	1.20 (0.64, 2.23) 0.574	90.1	1.13 (0.68, 1.88) 0.629	85.9	0.63 (0.22, 1.80) 0.390	85.1
GC	2	0.88 (0.42, 1.84) 0.738	95.1	0.92 (0.28, 3.04) 0.896	93.8	1.23 (0.97, 1.56) 0.093	0.0	1.00 (0.54, 1.84) 0.993	84.7	0.84 (0.27, 2.59) 0.756	94.5
BC	6	1.13 (0.87, 1.46) 0.351	89.5	1.33 (0.79, 2.25) 0.284	83.8	1.02 (0.74, 1.40) 0.908	85.5	1.09 (0.79, 1.52) 0.594	88.1	1.33 (0.85, 2.08) 0.211	80.8
CC	2	1.22 (0.78, 1.90) 0.379	82.4	1.46 (0.60, 3.55) 0.399	76.3	1.21 (0.80, 1.84) 0.364	62.5	1.26 (0.76, 2.07) 0.371	76.3	1.34 (0.66, 2.72) 0.416	65.8
OSCC	3	1.31 (1.14, 1.50) 0.000^*^	24.8	1.89 (1.18, 3.00) 0.008^*^	57.7	1.27 (1.07, 1.50) 0.006^*^	0.0	1.36 (1.16, 1.60) 0.000^*^	0.0	1.67 (1.04, 2.68) 0.035^*^	64.6
Cancer type											
LC	3	1.16 (1.06, 1.27) 0.002^*^	0.0	1.38 (1.14, 1.67) 0.001^*^	0.0	1.09 (0.95, 1.26) 0.219	0.0	1.16 (1.01, 1.33) 0.031	0.0	1.31 (1.03, 1.66) 0.028^*^	44.7
HCC	2	0.79 (0.60, 1.05) 0.100	71.7	0.68 (0.49, 0.93) 0.017^*^	0.0	0.77 (0.44, 1.34) 0.359	86.3	0.75 (0.47, 1.21) 0.237	83.8	0.73 (0.54, 1.00) 0.048^*^	0.0
rs2839698G/A											
Total	26	1.10 (1.01, 1.20) 0.039^*^	82.8	1.29 (1.09, 1.52) 0.003^*^	74.7	1.06 (0.97,1.17) 0.215	68.5	1.11 (1.01, 1.23) 0.036^*^	75.4	1.18 (1.01, 1.39) 0.042^*^	76.6
Ethnicity											
Caucasian	1	1.03 (0.78, 1.37) 0.827		1.10 (0.63, 1.92) 0.745		0.65 (0.40, 2.06) 0.084		0.78 (0.50, 1.22) 0.276		1.43 (0.90, 2.30) 0.134	
Asian	25	1.10 (1.00, 1.21) 0.041^*^	83.5	1.30 (1.09, 1.54) 0.003^*^	75.7	1.07 (0.98, 1.18) 0.138	68.2	1.12 (1.02, 1.24) 0.024^*^	75.9	1.17 (0.99, 1.39) 0.060	74.4
Source of control											
PB	5	1.06 (0.94, 1.21) 0.344	45.6	1.22 (0.95, 1.57) 0.122	34.4	0.94 (0.76,1.16) 0.560	55.2	1.00 (1.025,1.21) 0.983	51.3	1.28 (1.02, 1.59) 0.032^*^	27.9
HB	21	1.10 (0.99, 1.23) 0.072	85.3	1.30 (1.07, 1.59) 0.009^*^	78.5	1.09 (0.98,1.21) 0.106	69.5	1.14 (1.02, 1.28) 0.025^*^	77.6	1.16 (0.95, 1.04) 0.142	80.2
NOS scores											
N1	14	1.01 (0.90, 1.15) 0.830	76.6	1.14 (0.91, 1.43) 0.251	73.2	0.99 (0.88,1.11) 0.843	60.3	1.02 (0.91, 1.16) 0.704	66.4	1.02 (0.80, 1.31) 0.881	81.1
N2	12	1.20 (1.08, 1.34) 0.001^*^	82.0	1.46 (1.17, 1.81) 0.001^*^	69.4	1.14 (1.00, 1.29) 0.052	65.7	1.20 (1.05, 1.38) 0.010^*^	73.4	1.39 (1.17, 1.65) 0.000^*^	54.8
Sample size											
S1	10	1.07 (0.87, 1.32) 0.510	86.5	1.33 (0.92, 1.92) 0.134	79.6	1.11 (0.91,1.35) 0.317	67.9	1.17 (0.95,1.45) 0.138	75.3	1.08 (0.74, 1.57) 0.692	84.5
S2	14	1.11 (1.01, 1.21) 0.030^*^	79.7	1.28 (1.07, 1.53) 0.006^*^	71.6	1.04 (0.94, 1.16) 0.425	91.0	1.09 (0.97, 1.22) 0.137	77.2	1.25 (1.09, 1.45) 0.002^*^	60.7
Cancer type											
BLC	2	1.00 (0.89, 1.12) 0.992	0.0	1.03 (0.79, 1.36) 0.819	0.0	0.85 (0.59, 1.24) 0.405	58.0	0.96 (0.82, 1.13) 0.570	0.0	1.15 (0.84, 1.58) 0.379	28.0
GC	2	1.33 (1.13, 1.56) 0.000^*^	0.0	1.76 (1.26, 2.46) 0.001^*^	0.0	1.07 (0.75, 1.54) 0.699	52.3	1.27 (1.03, 1.57) 0.024^*^	0.0	1.74 (1.27, 2.40) 0.001^*^	0.0
HCC	3	1.15 (1.03, 1.29) 0.014^*^	0.0	1.33 (1.03, 1.72) 0.027^*^	0.0	1.12 (0.83, 1.51) 0.299	73.1	1.17 (0.95, 1.44) 0.136	45.4	1.27 (0.94, 1.73) 0.117	34.5
CRC	2	0.98 (0.65, 1.48) 0.927	90.9	1.01 (0.42, 2.42) 0.981	90.0	0.96 (0.64, 1.42) 0.817	79.6	0.95 (0.59, 1.55) 0.842	87.8	1.06 (0.54, 2.07) 0.869	85.0
LC	2	0.92 (0.81, 1.04) 0.178	0.0	0.78 (0.54, 1.13) 0.192	33.4	0.97 (0.78, 1.23) 0.822	31.0	0.93 (0.78, 1.09) 0.359	0.0	0.76 (0.47, 2.25) 0.283	63.4
BC	4	0.96 (0.63, 1.45) 0.839	94.3	1.72 (0.88, 3.34) 0.111	89.3	1.28 (0.86, 1.90) 0.220	87.3	1.40 (0.91, 1.11) 0.132	90.7	0.91 (0.45, 1.83) 0.789	92.7
rs3741219 A/G											
Total	10	1.07 (0.88, 1.30) 0.507	89.4	1.18 (0.94, 1.48) 0.154	61.8	0.97 (0.71, 1.33) 0.842	91.8	1.56 (0.79, 1.41) 0.709	91.8	1.14 (1.01, 1.29) 0.037^*^	0.0
Ethnicity											
Asian	10	1.07 (0.88, 1.30) 0.507	89.4	1.18 (0.94, 1.48) 0.154	61.8	0.97 (0.71, 1.33) 0.842	91.8	1.56 (0.79, 1.41) 0.709	91.8	1.14 (1.01, 1.29) 0.037^*^	0.0
Source of control											
PB	2	0.94 (0.85, 1.04) 0.253	0.0	0.96 (0.76, 1.22) 0.730	0.0	0.90 (0.79, 1.03) 0.126	0.0	0.91 (0.80, 1.04) 0.149	0.0	1.00 (0.80, 1.26) 0.978	0.0
HB	8	1.11 (0.86, 1.45) 0.424	91.6	1.28 (0.96, 1.71) 0.100	67.6	0.98 (0.63, 1.54) 0.941	93.6	1.10 (0.74, 1.64) 0.625	93.6	1.20 (1.04, 1.39) 0.014	0.0
NOS scores											
N1	5	1.07 (0.74, 1.55) 0.726	94.2	1.19 (0.79, 1.79) 0.417	78.9	0.88 (0.46, 1.67) 0.691	95.4	1.04 (0.60, 1.79) 0.898	95.4	1.10 (0.94, 1.30) 0.235	0.0
N2	4	1.11 (1.02, 1.21) 0.015^*^	20.0	1.26 (1.03, 1.53) 0.022^*^	0.0	1.10 (0.97, 1.24) 0.131	0.0	1.12 (1.00, 1.26) 0.042^*^	0.0	1.19 (0.98, 1.43) 0.038^*^	29.3
Sample size											
S1	5	0.94 (0.73, 1.22) 0.119	76.2	1.44 (0.90, 2.29) 0.126	59.1	1.20 (0.82, 1.77) 0.348	67.4	1.32 (0.89, 1.95) 0.164	76.0	1.23 (0.96, 1.59) 0.107	21.8
S2	5	1.26 (0.94, 1.69) 0.642	93.3	1.07 (0.83, 1.37) 0.618	64.7	0.83 (0.53, 1.29) 0.399	95.5	0.88 (0.59, 1.31) 0.527	95.2	1.11 (0.97, 1.28) 0.137	0.0
Cancer type											
BC	3	1.20 (0.78, 1.85) 0.405	87.2	1.51 (0.72, 3.18) 0.281	78.0	1.21 (0.65, 2.28) 0.546	87.8	1.27 (0.67, 2.40) 0.462	89.5	1.14 (0.91, 1.42) 0.258	23.9
rs3024270 C/G											
Total	12	1.04 (0.97, 1.12) 0.261	45.0	1.12 (1.01, 1.24) 0.025^*^	40.6	1.00 (0.91, 1.09) 0.926	34.1	1.03 (0.95, 1.12) 0.421	19.9	1.09 (0.95, 1.25) 0.228	65.3
Ethnicity											
Asian	12	1.04 (0.97, 1.12) 0.261	45.0	1.12 (1.01, 1.24) 0.025^*^	40.6	1.00 (0.91, 1.09) 0.926	34.1	1.03 (0.95, 1.12) 0.421	19.9	1.09 (0.95, 1.25) 0.228	65.3
Source of control											
PB	2	1.16 (0.75, 1.80) 0.494	86.5	1.42 (1.05, 1.93) 0.025^*^	84.2	1.01 (0.76, 1.35) 0.936	0.0	1.15 (0.88, 1.49) 0.309	0.0	1.30 (0.53, 3.21) 0.568	92.4
HB	10	1.03 (0.98, 1.09) 0.289	6.9	1.09 (0.98, 1.21) 0.112	7.2	0.99 (0.91, 1.09) 0.902	44.9	1.02 (0.94, 1.12) 0.610	28.4	1.06 (0.95, 1.17) 0.320	35.9
NOS scores											
N1	5	1.04 (0.96, 1.15) 0.336	0.0	1.10 (0.95, 1.27) 0.220	0.0	0.96 (0.84, 1.09) 0.502	68.6	1.00 (0.88, 1.13) 0.968	49.0	1.10 (0.98, 1.23) 0.117	0.0
N2	7	1.05 (0.93, 1.19) 0.457	68.2	1.14 (1.00, 1.31) 0.056	65.9	1.03 (0.92, 1.15) 0.644	0.0	1.06 (0.95, 1.19) 0.271	0.0	1.08 (0.85, 1.37) 0.550	78.6
Sample size											
S1	1	0.93 (0.73, 1.18) 0.545		0.88 (0.54, 1.43) 0.602		1.11 (0.72, 1.72) 0.625		1.02 (0.68, 1.54) 0.914		0.82 (0.56, 1.19) 0.295	
S2	11	1.05 (0.98, 1.13) 0.205	47.4	1.13 (1.02, 1.26) 0.017	42.9	0.99 (0.91, 1.08) 0.845	39.1	1.04 (0.95, 1.13) 0.424	27.1	1.11 (0.96, 1.28) 0.146	65.8
Cancer type											
BLC	2	1.07 (0.96, 1.19) 0.230	0.0	1.18 (0.94, 1.48) 0.151	0.0	1.17 (0.95, 1.45) 0.151	0.0	1.17 (0.96, 1.43) 0.124	0.0	1.05 (0.89, 1.22) 0.574	0.0
HCC	3	1.11 (0.84, 1.47) 0.452	84.9	1.20 (0.97, 1.48) 0.089	83.1	1.04 (0.86, 1.26) 0.711	44.7	1.10 (0.92, 1.31) 0.313	47.0	1.22 (0.73, 2.03) 0.448	89.2
rs3741216 A/T											
Total	4	1.66 (0.87, 3.18) 0.127	95.9	1.16 (0.85, 1.56) 0.348	0.0	0.91 (0.78, 1.06) 0.236	0.0	0.95 (0.82, 1.10) 0.471	0.0	2.42 (0.66, 8.83) 0.181	95.7
Ethnicity											
Asian	4	1.66 (0.87, 3.18) 0.127	95.9	1.16 (0.85, 1.56) 0.348	0.0	0.91 (0.78, 1.06) 0.236	0.0	0.95 (0.82, 1.10) 0.471	0.0	2.42 (0.66, 8.83) 0.181	95.7
Source of control											
HB	4	1.66 (0.87, 3.18) 0.127	95.9	0.87 (0.64, 1.17) 0.348	0.0	0.91 (0.78, 1.06) 0.236	0.0	0.95 (0.82, 1.10) 0.471	0.0	2.42 (0.66, 8.83) 0.181	95.7
NOS scores											
N1	2	2.96 (0.32, 27.18) 0.336		1.07 (0.65, 1.75) 0.798		0.97 (0.62, 1.50) 0.883		1.00 (0.67, 1.48) 0.981		4.22 (0.21, 84.51) 0.347	98.4
N2	2	0.99 (0.87, 1.14) 0.916	0.0	0.77 (0.52, 1.12) 0.170	0.0	1.12 (0.95, 1.32) 0.186	0.0	0.94 (0.80, 1.10) 0.444	0.0	1.35 (0.92, 1.97) 0.127	0.0
Sample size											
S1	2	2.96 (0.32, 27.18) 0.336		1.07 (0.65, 1.75) 0.798		0.97 (0.62, 1.50) 0.883		1.00 (0.67, 1.48) 0.981		4.22 (0.21, 84.51) 0.347	98.4
S2	2	0.99 (0.87, 1.14) 0.916	0.0	0.77 (0.52, 1.12) 0.170	0.0	1.12 (0.95, 1.32) 0.186	0.0	0.94 (0.80, 1.10) 0.444	0.0	1.35 (0.92, 1.97) 0.127	0.0
Cancer type											
GC	2	1.01 (0.84, 1.21) 0.944	0.0	1.09 (0.72, 1.64) 0.697	4.7	0.97 (0.75, 1.24) 0.779	0.0	0.99 (0.78, 1.25) 0.941	0.0	1.08 (0.65, 1.70) 0.748	22.9

BC: breast cancer; LC: lung cancer; BLC: bladder cancer; GC: gastric cancer; CRC: colorectal cancer; PC: pancreatic cancer; OC: ovarian cancer; CC: cervical cancer; OSCC: oral squamous cell carcinoma; UCC: urothelial cell carcinoma; RCC: renal cell carcinoma. ^*^P < 0.05

### Correlation between rs217727 G/A polymorphism and cancer risk

**Fig. 3:**
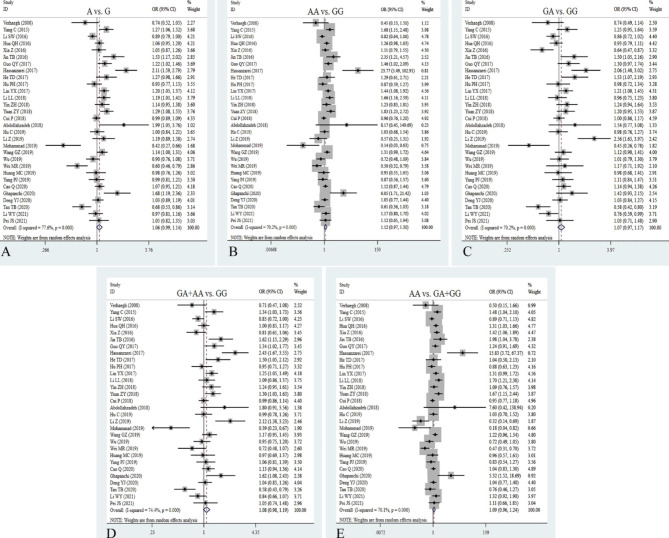
Forest plots for the association between H19 rs217727 polymorphism and cancer risk in five models. **A**: allele model; **B**: homozygote model; **C**: heterozygote model; **D**: dominant model; **E**: recessive model

Intriguingly, we obtained thirty studies about the relationship between rs217727 polymorphism and cancer risk with 14,215 patients and 20,247 controls. Overall, the rs217727 polymorphism was not significantly correlated with cancer risk (Table 2; Fig. 3). The allelic, homozygote, dominant and recessive models of rs217727 notably increased the risk of lung cancer (A vs. G: OR = 1.16, 95% CI = 1.06–1.27, *P* = 0.002; AA vs. GG: OR = 1.38, 95% CI = 1.14–1.67, *P* = 0.001; AA + GA vs. GG: OR = 1.16, 95% CI = 1.01–1.33, *P* = 0.031; AA vs. GG + GA: OR = 1.31, 95% CI = 1,03-1.66, *P* = 0.028) and oral squamous cell carcinoma (A vs. G: OR = 1.31, 95% CI = 1.14–1.50, *P* = 0.000; AA vs. GG: OR = 1,89, 95% CI = 1.18-3.00, *P* = 0.008; GA vs. GG: OR = 1.27, 95% CI = 1.07–1.50, *P* = 0.006; AA + GA vs. GG: OR = 1.36, 95% CI = 1.16–1.60, *P* = 0.000; AA vs. GG + GA: OR = 1.67, 95% CI = 1.04–2.68, *P* = 0.035, Table 2). Additionally, the rs217727 mutation significantly decreased the risk of hepatocellular carcinoma (GA vs. GG: OR = 0.68, 95% CI = 0.49–0.93, *P* = 0.017; AA vs. GG + GA: OR = 0.73, 95% CI = 0.54-1.00, *P* = 0.048, Table 2), suggesting that the rs217727 mutation may be an important protective factor for liver cancer, but a key risk factor for lung cancer and oral squamous cell carcinoma. The pooled results indicated that the homozygote and recessive models of rs217727 have a positive association with cancer risk in larger sample size (AA vs. GG: OR = 1.17, 95% CI = 1.02–1.33, *P* = 0.022; AA vs. GG + GA: OR = 1.14, 95% CI = 1.03–1.28, *P* = 0.015, Table 2). Heterogeneity was shown to exist in all five gene models, and results demonstrated that heterogeneity significantly decreased or disappeared in lung cancer and oral squamous cell carcinoma.

### Correlation between rs2839698 G/A polymorphism and cancer risk

**Fig. 4:**
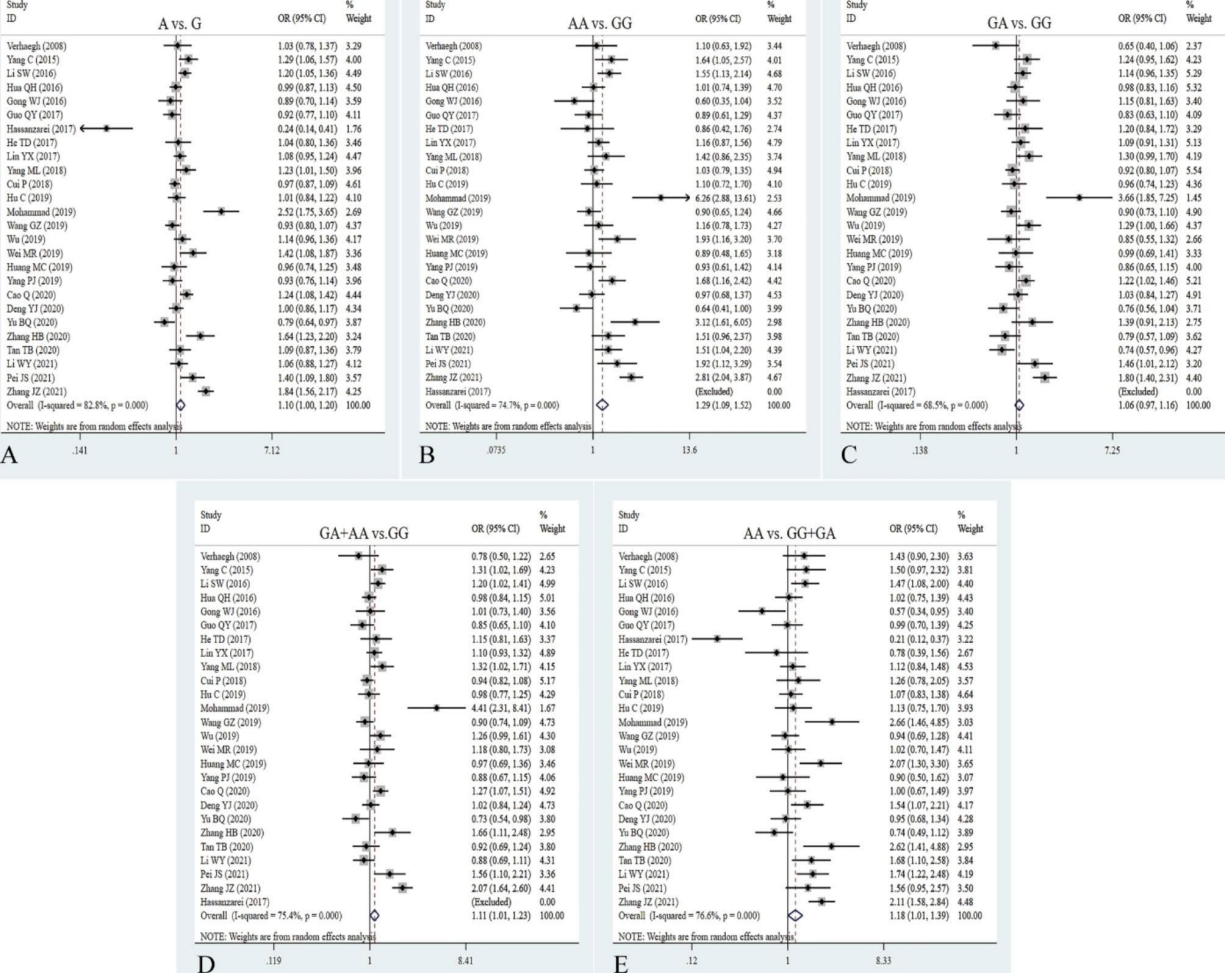
Forest plots for the association between H19 rs2839698 polymorphism and cancer risk in five models. **A**: allele model; **B**: homozygote model; **C**: heterozygote model; **D**: dominant model; **E**: recessive model

A total of twenty-six studies with 12,413 cancer patients and 18,650 controls were included to examine the association between H19 SNP rs2839698 and cancer risk. The rs2839698 polymorphism remarkably enhanced the risk of cancer in the allelic, homozygote, dominant and recessive models (A vs. G: OR = 1.10, 95% CI = 1.01–1.20, *P* = 0.039; AA vs. GG: OR = 1.29, 95% CI = 1.09–1.52, *P* = 0.003; AA + GA vs. GG: OR = 1.18, 95% CI = 1.01–1.23, *P* = 0.036; AA vs. GG + GA: OR = 1.18, 95% CI = 1.01–1.39, *P* = 0.042, Table 2; Fig. 4). Next, stratification analyses by cancer type showed the rs2839698 mutation significantly increased the risk of gastric cancer (A vs. G: OR = 1.33, 95% CI = 1.13–1.56, *P* = 0.000; AA vs. GG: OR = 1.76, 95% CI = 1.26–2.46, *P* = 0.001; AA + GA vs. GG: OR = 1.27, 95% CI = 1.03–1.57, *P* = 0.024; AA vs. GG + GA: OR = 1.74, 95% CI = 1.27–2.40, *P* = 0.001), hepatocellular cancer (A vs. G: OR = 1.17, 95% CI = 1.03–1.34, *P* = 0.015; GA vs. GG: OR = 1.30, 95% CI = 1.08–1.56, *P* = 0.006; AA + GA vs. GG: OR = 1.29, 95% CI = 1.08–1.93, *P* = 0.005), renal cell carcinoma and ovarian cancer, leukemia and lymphoma (Table 2). Similarly, a positive association was detected between the allelic, homozygous, and dominant models and cancer susceptibility in the Asian descent (A vs. G: OR = 1.10, 95% CI = 1.00-1.21, *P* = 0.041; AA vs. GG: OR = 1.30, 95% CI = 1.09–1.54, *P* = 0.003; AA + GA vs. GG: OR = 1.12, 95% CI = 1.02–1.24, *P* = 0.024, Table 2). When stratifying by source of control, quality score and sample size, the significantly increased cancer risk was discovered in hospital-based control (AA vs. GG: OR = 1.30, 95% CI = 1.07–1.59, *P* = 0.009; AA + GA vs. GG: OR = 1.14, 95% CI = 1.02–1.28, *P* = 0.025), population-based control (AA vs. GG + GA: OR = 1.28, 95% CI = 1.02–1.59, *P* = 0.032) and large simple size (A vs. G: OR = 1.11, 95% CI = 1.01–1.21, *P* = 0.030; AA vs. GG: OR = 1.28, 95% CI = 1.07–1.53, *P* = 0.006; AA vs. GG + GA: OR = 1.25, 95% CI = 1.09–1.45, *P* = 0.002, Table 2). Heterogeneity results suggested that heterogeneity consisted in the five genetic models of overall analysis. Interestingly, we found that heterogeneity notably diminish or disappear in hepatocellular carcinoma, bladder, gastric, and lung cancer.

### Correlation between rs3741219 A/G polymorphism and cancer risk

**Fig. 5:**
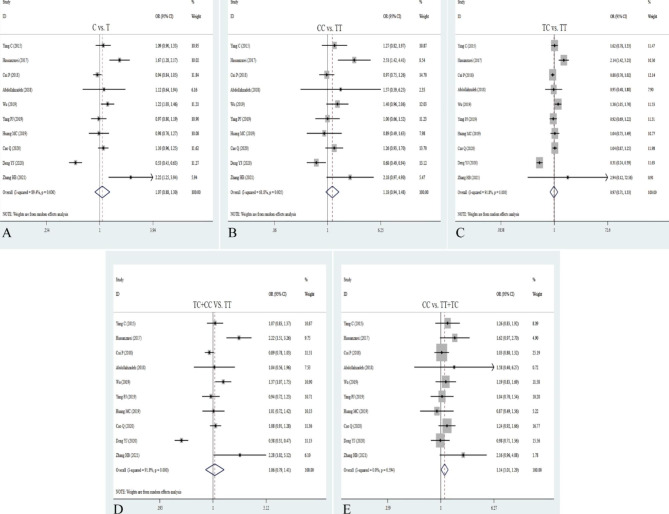
Forest plots for the association between H19 rs3741219 polymorphism and cancer risk in five models. **A**: allele model; **B**: homozygote model; **C**: heterozygote model; **D**: dominant model; **E**: recessive model

To explore the association between H19 rs3741219 polymorphism and cancer risk, we included 10 studies with 5305 patients and 6974 controls. Compared with AA + GA genotype, the GG allele of rs3741219 polymorphism was correlated with cancer susceptibility in overall analysis (AA vs. GG + GA: OR = 1.14, 95% CI = 1.01–1.29; *P* = 0.037, Table 2; Fig. 5). Stratified analyses indicated that the rs3741219 mutant remarkably enhanced the risk of hepatocellular carcinoma and ovarian cancer, but also decreased the risk of Glioma tumor. We next performed stratification analysis by source of control and sample size, the pooled results indicated no association between 3,741,219 polymorphism and cancer risk. Beyond that, subgroup analyses by quality score strongly showed an elevated cancer risk in higher quality score (G vs. A: OR = 1.11, 95% CI = 1.02–1.21, *P* = 0.015; GG vs. AA: OR = 1.26, 95% CI = 1.03–1.53, *P* = 0.022; GG + GA vs. AA: OR = 1.12, 95% CI = 1.00-1.26, *P* = 0.042; GG vs. AA + GA: OR = 1.19, 95% CI = 0.98–1.43, *P* = 0.038, Table 2). It manifested that heterogeneity mainly appeared in the five gene models of overall analysis and Asian population. Moreover, there was no heterogeneity existing in population-based and higher quality score.

### Correlation between rs3024270 C/G polymorphism and cancer risk

**Fig. 6:**
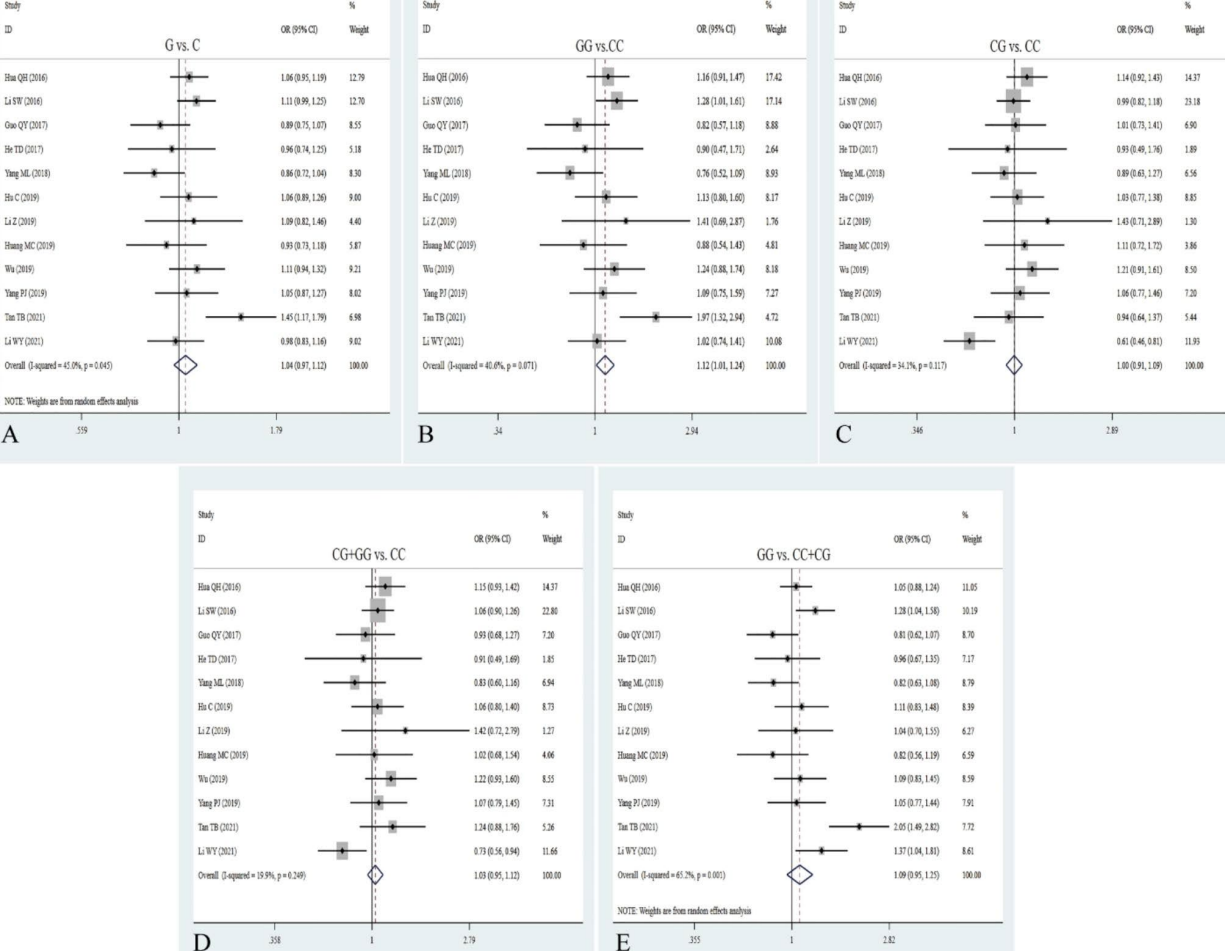
Forest plots for the association between H19 rs3024270 polymorphism and cancer risk in five models. **A**: allele model; **B**: homozygote model; **C**: heterozygote model; **D**: dominant model; **E**: recessive model

Through integrating 12 potential studies embodying 5402 patients and 9159 controls, we found a significant relationship of rs3024270 polymorphism with cancer risk under homozygous model (GG vs. CC: OR = 1.12, 95% CI = 1.01–1.24, P = 0.025, Table 2; Fig. 6). The homozygous and recessive models of rs3024270 were significantly correlated with the increased risk of colorectal cancer (GG vs. CC: OR = 1.28, 95% CI = 1.01–1.61, P = 0.041; GG vs. CC + GC: OR = 1.29, 95% CI = 1.04–1.58, P = 0.019). There was no significant association between the rs3024270 polymorphism and cancer susceptibility in stratification analysis by ethnicity and quality score. We found that the rs3024270 polymorphism was positively related to cancer risk in hospital-based controls under the homozygote model (GG vs. CC: OR = 1.42, 95% CI = 1.05–1.93; *P* = 0.025, Table 2). Except for the recessive model (*I*^*2*^ = 65.3%, *P* = 0.001), there was no heterogeneity in other models.

### Correlation between rs3741216 A/T polymorphism and cancer risk

**Fig. 7:**
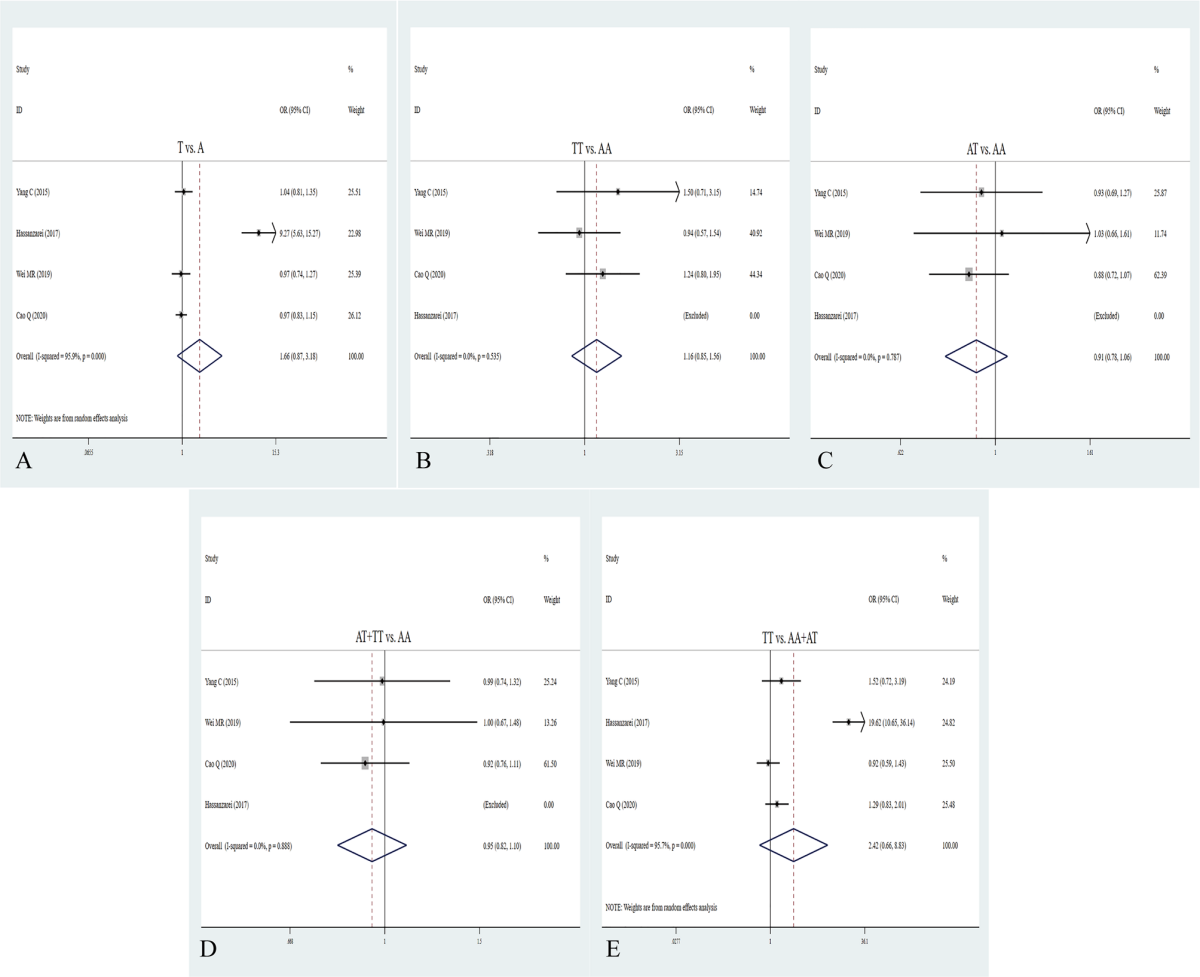
Forest plots for the association between H19 rs3741216 polymorphism and cancer risk in five models. **A**: allele model; **B**: homozygote model; **C**: heterozygote model; **D**: dominant model; **E**: recessive model

In general, four eligible studies with 2049 patients and 1808 controls were included to detect the relation between rs2107425 polymorphism and cancer risk. The pooled results suggested that the rs2107425 polymorphism was not related to cancer risk in five genetic models (T vs. A: OR = 1.66, 95% CI = 0.87–3.18, *P* = 0.127; TT vs. AA: OR = 1.66, 95% CI = 0.85–1.56, *P* = 0.348; AT vs. AA: OR = 0.91, 95% CI = 0.78–1.06, *P* = 0.236; AT + TT vs. AA: OR = 0.95, 95% CI = 0.82–1.10, *P* = 0.471; TT vs. AA + AT: OR = 2.42, 95% CI = 0.66–8.83, *P* = 0.181, Table 2; Fig. 7). Similarly, when stratifying analyses by ethnicity, cancer type, quality score, and source of control, we did find any correlation between the rs3741216 polymorphism and cancer risk. The result of heterogeneity test exhibited *I*^*2*^ = 95.9 and 95.7, implying that heterogeneity clearly exists in both homologous and recessive models, and thus random-effects model was used to examine the correlation.

### FPRP results

We investigated determinants of FPRP across a range of probabilities to determine whether a given association between *H19* SNPs and cancer risk is deserving of attention or is noteworthy. In this respect, we found that our main results were further supported by FPRP analysis. As shown in Table 3, with a prior probability < 0.25, the *H19* rs2839698 polymorphism was associated with the risk of cancer under allele, homozygote, dominant and recessive models. Similarly, with a prior probability of 0.25, the homozygote model of *H19* rs3024270 polymorphism was associated with cancer risk and the recessive model of *H19* rs3024270 polymorphis was associated with cancer risk (*P* < 0.2).

**Table 3:** False-positive report probability analysis of the noteworthy results

					Prior probability				
SNP	Genetic model	OR (95% CI)	P	Power	0.25	0.1	0.01	0.001	0.0001
rs2107425	Allele	0.98 (0.91, 1.06)	0.614	1.000	0.648	0.847	0.984	0.998	1.000
	Homozygote	1.01 (0.88, 1.17)	0.894	1.000	0.729	0.890	0.989	0.999	1.000
	Heterozygote	0.96 (0.85, 1.07)	0.461	1.000	0.580	0.806	0.979	0.998	1.000
	Dominant	0.97 (0.87, 1.08)	0.578	1.000	0.634	0.839	0.983	0.998	1.000
	Recessive	0.98 (0.91, 1.06)	0.578	1.000	0.648	0.847	0.984	0.998	1.000
rs217727	Allele	1.06 (0.99, 1.14)	0.116	1.000	0.259	0.512	0.920	0.991	0.999
	Homozygote	1.12 (0.97, 1.30)	0.136	1.000	0.290	0.551	0.931	0.933	0.999
	Heterozygote	1.07 (0.97, 1.17)	0.138	1.000	0.292	0.554	0.932	0.993	0.999
	Dominant	1.08 (0.98, 1.19)	0.120	1.000	0.265	0.519	0.922	0.992	0.999
	Recessive	1.09 (0.96, 1.24)	0.191	1.000	0.363	0.631	0.950	0.995	0.999
rs2839698	Allele	1.10 (1.01, 1.20)	0.032	1.000	0.087^*^	0.223	0.759	0.969	0.997
	Homozygote	1.29 (1.09, 1.52)	0.002	0.964	0.007^*^	0.021	0.194	0.709	0.961
	Heterozygote	1.06 (0.97,1.17)	0.247	1.000	0.426	0.690	0.961	0.996	1.000
	Dominant	1.11 (1.01, 1.23)	0.046	1.000	0.122^*^	0.294	0.821	0.979	0.998
	Recessive	1.18 (1.01, 1.39)	0.048	0.998	0.125^*^	0.300	0.825	0.979	0.998
rs3741219	Allele	1.07 (0.88, 1.30)	0.500	1.000	0.598	0.817	0.980	0.998	1.000
	Homozygote	1.18 (0.94, 1.48)	0.152	0.981	0.317	0.583	0.939	0.994	0.999
	Heterozygote	0.97 (0.71, 1.33)	0.850	0.999	0.720	0.885	0.988	0.999	1.000
	Dominant	1.06 (0.79, 1.41)	0.689	0.991	0.674	0.861	0.986	0.999	1.000
	Recessive	1.14 (1.01, 1.29)	0.038	1.000	0.102^*^	0.254	0.789	0.974	0.997
rs3024270	Allele	1.04 (0.97, 1.12)	0.300	1.000	0.473	0.729	0.967	0.997	1.000
	Homozygote	1.12 (1.01, 1.24)	0.029	1.000	0.080^*^	0.207	0.742	0.967	0.997
	Heterozygote	1.00 (0.92, 1.09)	0.928	1.000	0.736	0.893	0.989	0.999	1.000
	Dominant	1.03 (0.95, 1.12)	0.489	1.000	0.595	0.815	0.980	0.998	1.000
	Recessive	1.09 (0.95, 1.25)	0.228	1.000	0.406	0.673	0.958	0.996	1.000
rs3741216	Allele	1.66 (0.87, 3.18)	0.126	0.380	0.500	0.750	0.971	0.997	1.000
	Homozygote	1.16 (0.85, 1.56)	0.773	0.691	0.731	0.891	0.989	0.999	1.000
	Heterozygote	0.91 (0.78, 1.06)	0.226	1.000	0.404	0.670	0.957	0.996	1.000
	Dominant	0.95 (0.82, 1.10)	0.493	1.000	0.597	0.816	0.980	0.998	1.000
	Recessive	2.42 (0.66, 8.83)	0.181	0.234	0.689	0.874	0.987	0.999	1.000

^*^P < 0.2

### Sensitivity analysis and publication bias

**Fig. 8:**
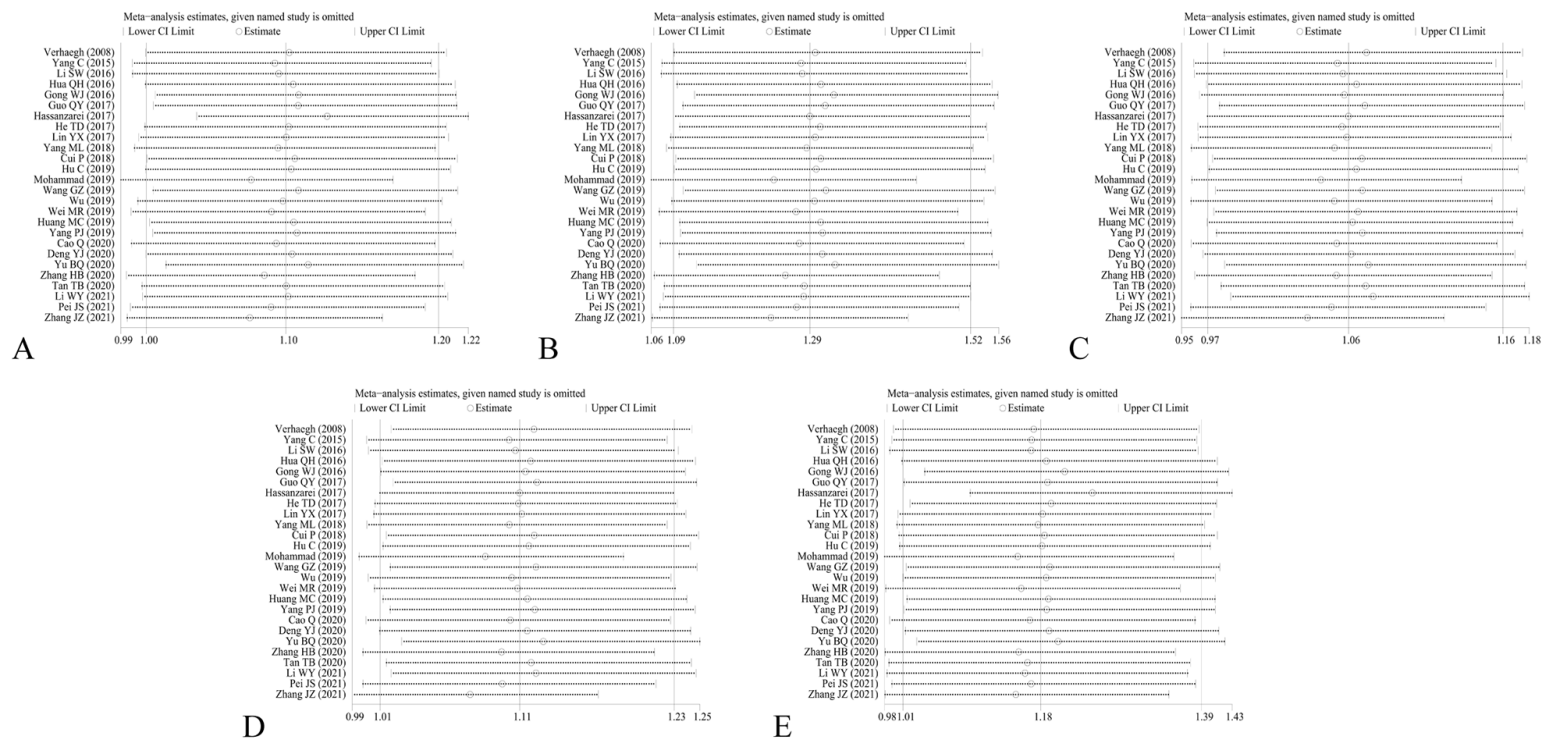
Sensitivity analysis for H19 rs2839698 polymorphism and cancer risk in five models. **A**: allele model; **B**: homozygote model; **C**: heterozygote model; **D**: dominant model; **E**: recessive model

**Fig. 9:**
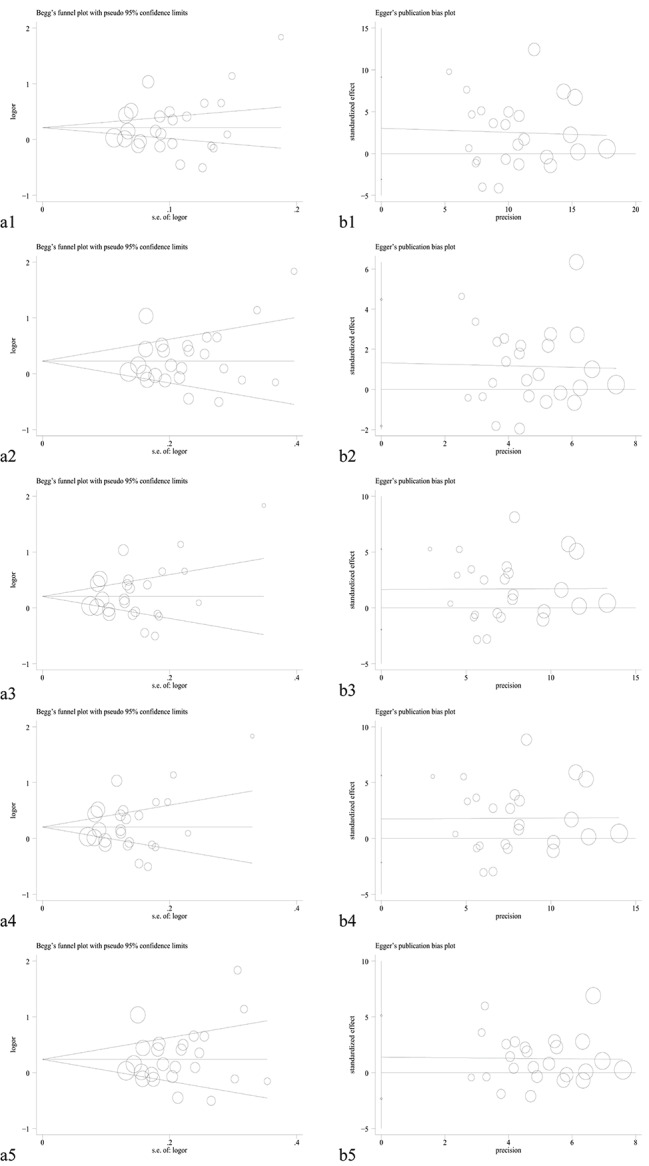
Begg’s funnel plot and Egger’s linear regression plot for detecting the publication bias in rs2839698 polymorphism. (**a1**) Begg’s funnel plot and (**b1**) Egger’s linear regression plot in the allele model; (**a2**) Begg’s funnel plot and (**b2**) Egger’s linear regression plot in the homozygote model; (**a3**) Begg’s funnel plot and (**b3**) Egger’s linear regression plot in the homozygote model; (**a4**) Begg’s funnel plot and (**b4**) Egger’s linear regression plot in the heterozygote model; (**a5**) Begg’s funnel plot and (**b5**) Egger’s linear regression plot in the recessive model

Sensitivity analysis was conducted by eliminating each individual study. As shown in Fig. 8, the pooled OR and 95% CI were not materially changed, indicating that our results were relatively robust. After excluding several studies inconsistent with HWE, we found substantial alteration under the allele and heterozygous models in rs3741216 polymorphism (allelic: I^2^ = 0.0%, P_(heterogeneity)_ = 0.649; heterozygous: I^2^ = 0.0, P_(heterogeneity)_ = 0.678; homozygous: I^2^ = 0.0%, P_(heterogeneity)_ = 0.737; dominant: I^2^ = 0.0%, P_(heterogeneity)_ = 0.681; recessive: I^2^ = 0.0%, P_(heterogeneity)_ = 0.708, Table 4). Other three gene polymorphisms were not substantially changed. In addition, funnel plot was symmetrical, and no evident publication bias was observed by using the Begg’s test and Egger’s test (Table 5; Fig. 9).

**Table 4:** After excluding studies inconsistent with HWE, the associations between four H19 polymorphisms and cancer risk under five genetic models

Genetic model		rs217727G/A	rs2839698G/A	rs3741219T/C	rs3741216A/T
Allele	OR (95%CI) *P* I^2^ (%) *P*_*(heterogeneity)*_	1.06 (1.00, 1.13) 0.071 69.8 0.000	1.06 (0.98, 1.15) 0.132 77.1 0.000	1.02 (0.84, 1.24) 0.850 89.6 0.000	0.99 (0.87, 1.14) 0.916 0.0 0.649
Homozygote	OR (95%CI) *P* I^2^ (%) *P*_*(heterogeneity)*_	1.13 (1.00, 1.28) 0.053 60.6 0.000	1.21 (1.04, 1.41) 0.014 66.2 0.000	1.14 (0.91, 1.43) 0.269 61.6 0.000	0.31 (0.89, 1.92) 0.170 0.0 0.678
Heterozygote	OR (95%CI) *P* I^2^ (%) *P*_*(heterogeneity)*_	1.04 (0.95, 1.14) 0.369 68.2 0.000	1.04 (0.95, 1.13) 0.392 61.3 0.000	0.96 (0.70, 1.32) 0.795 92.7 0.000	0.89 (0.76, 1.06) 0.186 0.0 0.737
Dominant	OR (95%CI) *P* I^2^ (%) *P*_*(heterogeneity)*_	1.67 (0.98, 1.17) 0.157 70.3 0.000	1.07 (0.98, 1.17) 0.124 66.6 0.000	1.00 (0.75, 1.35) 0.978 92.4 0.000	0.94 (0.80, 1.10) 0.444 0.0 0.681
Recessive	OR (95%CI) *P* I^2^ (%) *P*_*(heterogeneity)*_	1.11 (0.99, 1.24) 0.073 59.2 0.000	1.12 (0.96, 1.32) 0.147 72.9 0.000	1.12 (0.99, 1.27) 0.074 0.0 0.763	1.35 (0.92, 1.97) 0.127 0.0 0.708

**Table 5:** Publication bias of the five genetic models for H19 gene polymorphisms

Variables	Allelic		Homozygous		Heterozygous		Dominant		Recessive
	P _B_ P_E_		P _B_ P_E_		P _B_ P_E_		P _B_ P_E_		P _B_ P_E_
rs2107425C/T	0.360 0.336		0.583 0.436		0.246 0.286		0.300 0.287		0.583 0.532
rs217727G/A	0.454 0.515		0.592 0.494		0.475 0.489		0.354 0.445		0.748 0.540
rs2839698G/A	0.252 0.317		0.315 0.393		0.338 0.351		0.388 0.363		0.252 0.448
rs3741219A/G	0.371 0.404		0.371 0.265		0.592 0.571		0.371 0.346		0.474 0.311
rs3024270C/G	1.000 0.867		1.000 0.875		0.876 0.861		0.876 0.775		0.533 0.791
rs3741216A/T	0.308 0.170		1.000 0.200		1.000 0.815		1.000 0.815		1.000 0.803

P _B_: P-value of Begg’s rank correlation test. ^*^P < 0.05. P_E_: P-value of Egger’s linear regression test. ^*^P < 0.05

## Discussion

Cancer is one of the leading causes of mortality, seriously affecting public health all over the world. However, the pathogenesis of cancer remains poorly explicit. It is widely accepted that cancer may be influenced by genetic mutations [81]. As a newly identified non-coding RNAs, lncRNAs are extensively present in human genome. Numerous studies have confirmed that lncRNAs play essential roles in diverse biological activities, such as cell cycle processes, epigenetic regulation, transcriptional regulation, stress response and pluripotency maintenance [16, 18]. A large number of SNPs located in the lncRNAs may affect gene expression and function by altering its secondary structure or the targeted microRNAs, ultimately, leading to the occurrence and progression of cancer [82–84].

H19 belongs to a class of maternally expressed lncRNA at 2.3 kb length, which is located at imprinted region on chromosome 11p15.5. Differentially methylated region (DMR) usually refers to the upstream of the transcription initiation site of H19, which servers a vital role in the regulation of H19/IGF2 expression [85, 86]. It has been reported that H19 expression is prominently decreased after birth, and only exhibits in cardiac and skeletal muscles [82]. Accumulating evidence has shown that H19 gene polymorphisms are linked to cancer risk. Verhaegh et al. first reported that H19 rs2839698 variants significantly reduced the risk of bladder cancer among Caucasians, especially in non-muscle invasive bladder cancer [41]. Also, some studies have reported that the rs3741219 polymorphism was not associated with the risk of cancer, the rs2839698 polymorphism significantly increases the risk of gastrointestinal cancer, and the rs2107425 polymorphism had a protective effect on Caucasian population [81, 87, 88]. In order to accurately assess the association between H19 polymorphisms and the risk of cancer, we conducted a comprehensive analysis of all relevant potential studies.

Our findings suggested that the rs2839698, rs3741219 and rs3024270 polymorphisms, but not rs2107425, rs217727, or rs3741216 polymorphisms were associated with cancer risk in overall analysis. Among these, the rs2839698 polymorphism was dramatically related to increased cancer risk among Asians, while the rs210742 polymorphism was significantly associated with reduced cancer risk among Caucasians, indicating that ethnic differences in genetic backgrounds might influence the correlation. Using the RNA secondary structure prediction website, Gong et al. verified that the rs2107425 variant changed the minimum free energy of its centroid secondary structure and increased genetic susceptibility to cancer by impacting the H19 function and stability [51]. Further experimental functional studies are necessary to prove the exact mechanism. We found that rs2839698 SNP was positively associated with cancer susceptibility among Asians.

In the present study, the rs217727 mutation positively associated with increased risk of oral squamous cell carcinoma and lung cancer, but reduced the risk of hepatocellular carcinoma. Moreover, the rs2839698 polymorphism was significantly correlated with increased risk of gastric cancer, which was consistent with a previous study [88]. There were significant correlations between the rs2839698 polymorphism and cancer risk in hospital-based control, sample size and quality score subgroups. These results provided evidence that rs2839698 could modify cancer susceptibility based on ethnicity and cancer type. Furthermore, the discrepancy between our results and previous studies may be attributed to different genetic backgrounds and sample sizes. As for the rs3741219 polymorphism, a marginally notable correlation was discovered under recessive model in overall analysis. The positive results of higher quality score showed remarkable association with rs3024270 polymorphism. Moreover, we did not observe any relationships between rs3741216 rs3024270 and cancer in overall and subgroup analyses.

Among these H19 SNPs, rs217727, rs2839698, rs3741219, and rs3741216 located in exon region, as well as rs3024270 in intron region. SNPs at exon region are more likely to alter the H19 conserved folding structure or complementary sequences to target genes, and thus modify its binding affinity with interacting elements, while SNPs at intronic region may participate in alternative splicing and regulation of H19 transcript [86, 89]. Li et al. found that genetic variants of rs2839689, rs217727, rs2735971 and rs3024270 were closely associated with changes of H19 secondary structure in colorectal cancer [57]. It has been reported that the rs217727 polymorphism affected interactions between miRNAs and H19 and induced formation of target miRNA sites, such as hsa-miR-4804-5p and hsa-miR-8071, leading to the loss of hsa-miR-3960 and hsa-miR-8071 binding sites [73]. In addition, the rs2839698 mutation causes the loss of hsa-miR-24-1-5p and hsa-miR-24-2-5p, hsa-miR-566, and miR-675 [71, 75]. We speculated that the rs2839698 variation might hinder the binding of these targeted miRNA sites to the H19 3’-UTR, and then disrupt the reciprocal repression-regulatory loop between them, resulting in the tumorigenesis and progression.

Several limitations should be taken into account in the present study. First, heterogeneities were observed in most of the H19 SNPs, and subgroup analyses by source of control, cancer type, and ethnic diversity failed to completely eliminate these heterogeneities. Second, with regard to rs3741219, rs3024270 and rs3741216 polymorphism, all the included subjects were from Asian, and except for one study from Caucasians in rs217727 and rs2839698 polymorphism, other studies were involved with Asian population, which may not be applicable to other populations. Third, each type of cancer with only one study was assigned to subgroup analysis by other cancers, and the number of included studies for certain H19 polymorphisms was relatively limited in the subgroup analysis. Finally, due to the lack of available data on some factors such as alcohol consumption, smoking, lifestyle and effects of haplotype, we cannot evaluate the impact of gene-environmental and gene-gene interactions.

## Conclusions

In conclusion, this meta-analysis demonstrated significant associations between H19 rs2839698 and rs3024270 and overall cancer risk. We found that H19 rs2107425 may be a protective factor for the Caucasian population, while rs2839698 may be a hazard factor for the Asian descent. Therefore, studies with larger sample sizes, diverse races and different cancer types are needed to further and better validate our findings.

**Abbreviations**: BC = breast cancer, LC = lung cancer, BLC = bladder cancer, GC = gastric cancer, CRC = colorectal cancer, PC = pancreatic cancer, OC = ovarian cancer, HCC: hepatocellular carcinoma, CC = cervical cancer, OSCC = oral squamous cell carcinoma, UCC = urothelial cell carcinoma, RCC = renal cell carcinoma, SNP = single nucleotide polymorphism, CI = confidence interval, HWE = Hardy-Weinberg equilibrium, NOS = Newcastle Ottawa Scale; OR = odds ratio.

## Electronic supplementary material

Below is the link to the electronic supplementary material.

Supplementary Material 1

Supplementary Material 2

Supplementary Material 3

Supplementary Material 4

## Data Availability

The datasets used and/or analyzed during the current study are available from the corresponding author on reasonable request.
