# Supplementary material for: Six polymorphisms in the lncRNA H19 gene and the risk of cancer: a systematic review and meta-analysis
Source: BMC Cancer. 2023 Jul 21;23:688. doi: 10.1186/s12885-023-11164-y (PMC10362596; doi:10.1186/s12885-023-11164-y)
Supplement: Supplementary file 1 — Supplementary Material 1 [file 12885_2023_11164_MOESM1_ESM.doc]

**Supplementary table 1.** Genotype frequency distributions of H19 SNPs in the present study.

| Authors | Years | Type of cancer | Country | Ethnicity | Source of control | Genotyping | SNP |
| --- | --- | --- | --- | --- | --- | --- | --- |
| Verhaegh[27] | 2008 | BLC | Netherlands | Caucasian | PB | PCR-RFLP | rs2107425C/T  rs217727C/T  rs2839698T/C  rs2735469C/T rs17658052G/A |
| Song HL[28] | 2009 | OC | Mixed | Caucasian | PB | TaqMan | rs2107425C/T |
| Quaye[29] | 2009 | OC | Mixed | Caucasian | PB | TaqMan | rs2107425C/T |
| Barnholtz[30] | 2010 | BC | USA | African | PB | Illumina | rs2107425C/T |
| Barnholtz[30] | 2010 | BC | USA | Caucasian | PB | Illumina | rs2107425C/T |
| Butt S[31] | 2012 | BC | Sweden | Caucasian | PB | MassArray | rs2107425C/T |
| Yang C[32] | 2015 | GC | China | Asian | HB | TaqMan | rs217727C/T  rs2839698C/T  rs3741216A/T  rs3741219T/C |
| Li SW[33] | 2016 | CRC | China | Asian | HB | TaqMan | rs2839698 G/A  rs3024270 C/G  rs217727 G/A  rs2735971C/T |
| Hua QH[34] | 2016 | BLC | China | Asian | HB | TaqMan | rs217727G/A  rs2735971C/T  rs2839698G/A  rs3024270G/C |
| Xia Z[35] | 2016 | BC | China | Asian | HB | PCR-RFLP | rs374129T/C  rs217727C/T |
| Jin TB[36] | 2016 | CC | China | Asian | HB | MassArray | rs217727C/T |
| Gong WJ[37] | 2016 | LC | China | Asian | HB | MassArray | rs2839698G/A  rs2107425C/T |
| Guo QY[38] | 2017 | OSCC | China | Asian | HB | Illumina | rs2735971C/T  rs217727G/A  rs2839698G/A  rs3024270G/C |
| Hassanzarei[39] | 2017 | BC | Iran | Asian | HB | PCR-RFLP | rs3741219T/C  rs217727C/T  rs2839698T/C  rs3741216T/A |
| He TD[40] | 2017 | OS | China | Asian | HB | TaqMan | rs2735971C/T  rs217727G/A  rs2839698G/A  rs3024270G/C |
| Hu PH[41] | 2017 | PC | China | Asian | HB | TaqMan | rs217727C/T |
| Lin YX[42] | 2017 | BC | China | Asian | HB | G0104K | rs217727C/T  rs2839698C/T |
| Li LL[43] | 2018 | LC | China | Asian | HB | TaqMan | rs217727C/T |
| Yang ML[44] | 2018 | HCC | China | Asian | HB | KASP | rs2735971G/A  rs2839698C/T  rs3024270G/C |
| Yin ZH[45] | 2018 | LC | China | Asian | HB | Illumina | rs217727C/T  rs2107425C/T  rs2735469C/T |
| Yin ZH[45] | 2018 | LC | China | Asian | HB | Illumina | rs17658052G/A |
| Yuan ZY[46] | 2018 | OSCC | China | Asian | HB | MassArry | rs217727C/T;  rs2839701C/G |
| Cui P[47] | 2018 | BC | China | Asian | HB | TaqMan | rs217727 G/A  rs2071095C/A  rs2251375C/A  rs2839698G/A  rs2839701C/G  rs3741219A/G  rs4930098G/C |
| Abdollahzadeh[48] | 2018 | BC | Iran | Asian | HB | PCR-RFLP | rs217727C/T  rs3741219T/C |
| Hu C[49] | 2019 | NB | China | Asian | HB | TaqMan | rs2839698G/A  rs3024270C/G  rs217727 G/A |
| Li Z[50] | 2019 | OSCC | China | Asian | HB | TaqMan | rs217727 G/A  rs2735971C/T  rs3024270G/C  rs2839698C/T  rs217727 C/T |
| Safari[51] | 2019 | BC | Iran | Asian | HB | ARMS-PCR | rs2839698C/T  rs217727 C/T |
| Yang PJ[52] | 2019 | UCC | China | Asian | HB | PCR | rs217727C/T  rs2107425C/T  rs2839698C/T  rs3024270C/G  rs3741219A/G |
| Wang GZ[53] | 2019 | LC | Iran | Asian | HB | TaqMan | rs2067051G/A;  rs217727C/T;  rs2839698C/T;  rs4929984C/A |
| Wu[54] | 2019 | HCC | China | Asian | HB | TaqMan | rs17658052G/A rs2107425C/T  rs2839698C/T  rs3024270C/T  rs3741219A/G |
| Wei MR[55] | 2019 | GC | China | Asian | HB | TaqMan | rs217727C/T  rs2735971T/C  rs2839698T/C  rs3741216A/T |
| Huang MC[56] | 2019 | CC | China | Asian | HB | PCR | rs302470C/G  rs2839698C/T  rs3741216A/T  rs2107425C/T  rs217727C/T |
| Cao Q[57] | 2020 | RCC | China | Asian | HB | RT-PCR | rs2839698 C/T  rs217727C/T  rs3741216A/T  rs3741219T/C |
| Ghapanchi[58] | 2020 | OSCC | Iran | Asian | HB | ARMS-PCR | rs217727C/T;  rs2107425C/T |
| Yu BQ[59] | 2020 | CRC | China | Asian | HB | PCR | rs2839698G/A |
| Deng YJ[60] | 2020 | Glioma | China | Asian | HB | MassArray | rs217727G/A  rs2839698G/A |
| Deng YJ[60] | 2020 | Glioma | China | Asian | HB | MassArray | rs3741219A/G |
| Zhang HB[61] | 2020 | OC | China | Asian | HB | MassArray | rs2525885T/C  rs2839698 G/A  rs3741206T/C  rs3741219G/A |
| Tan TB[62] | 2020 | HBa | China | Asian | HB | TaqMan | rs2839698G/A  rs3024270C/G  rs217727G/A |
| Li WY[63] | 2021 | Wilms | China | Asian | HB | TaqMan | rs2839698G/A  rs3024270C/G  rs217727G/A |
| Pei JS[64] | 2021 | Leukemia | China | Asian | HB | RT-PCR | rs2839698C/T  rs217727C/T |
| Zhang JZ[65] | 2021 | Lymphoma | Iran | Asian | HB | PCR-RFLP | rs2839698G/A |
| Khalil[66] | 2022 | CRC | Egypt | Asian | HB | QIAamp | rs2107425C/T |

BC: breast cancer; LC: lung cancer; BLC: bladder cancer; GC: gastric cancer; CRC: colorectal cancer; PC: pancreatic cancer; OC: ovarian cancer; CC: cervical cancer; OSCC: oral squamous cell carcinoma; UCC: urothelial cell carcinoma; RCC: renal cell carcinoma. HBa: Hepatoblastoma; OS: osteosarcoma; NB: Neuroblastoma.
