# Supplementary material for: Six polymorphisms in the lncRNA H19 gene and the risk of cancer: a systematic review and meta-analysis
Source: BMC Cancer. 2023 Jul 21;23:688. doi: 10.1186/s12885-023-11164-y (PMC10362596; doi:10.1186/s12885-023-11164-y)
Supplement: Supplementary file 2 — Supplementary Material 2 [file 12885_2023_11164_MOESM2_ESM.doc]

**Supplementary table 2.** NOS for VDR gen polymorphisms in CRC.

| Author | Year | Selection | | | |  | Comparability |  | Exposure | | | NOS |
| --- | --- | --- | --- | --- | --- | --- | --- | --- | --- | --- | --- | --- |
|  |  | Adequate definition of case | Representative of the cases | Selection of controls | Definition of controls |  | Comparability of cases and controls on the basis of the design or analysis |  | Ascertainment of exposure | Same method of ascertainment for cases and controls | Non-response rates |  |
| Verhaegh | 2008 | ☆ | ☆ | ☆ | ☆ |  | ☆☆ |  | ☆ | ☆ | ☆ | 9 |
| Song HL | 2009 | ☆ | ☆ | ☆ | ☆ |  | ☆☆ |  | ☆ | ☆ | ☆ | 9 |
| Quaye | 2009 | ☆ | ☆ | ☆ | ☆ |  |  |  | ☆ | ☆ |  | 6 |
| Barnholtz | 2010 | ☆ | ☆ | ☆ | ☆ |  | ☆ |  | ☆ | ☆ |  | 7 |
| Butt S | 2012 | ☆ | ☆ | ☆ | ☆ |  | ☆☆ |  |  |  |  | 8 |
| Yang C | 2015 | ☆ | ☆ |  | ☆ |  | ☆☆ |  | ☆ | ☆ | ☆ | 8 |
| Li SW | 2016 | ☆ | ☆ | ☆ | ☆ |  | ☆☆ |  | ☆ | ☆ | ☆ | 9 |
| Hua QH | 2016 | ☆ | ☆ |  | ☆ |  | ☆☆ |  | ☆ | ☆ |  | 7 |
| Xia Z | 2016 | ☆ | ☆ | ☆ | ☆ |  | ☆☆ |  | ☆ | ☆ | ☆ | 9 |
| Jin TB | 2016 | ☆ | ☆ | ☆ | ☆ |  | ☆☆ |  | ☆ | ☆ | ☆ | 9 |
| Gong WJ | 2017 | ☆ | ☆ |  | ☆ |  | ☆ |  | ☆ | ☆ |  | 6 |
| Guo QY | 2017 | ☆ | ☆ |  | ☆ |  | ☆☆ |  | ☆ | ☆ |  | 8 |
| Hassanzarei | 2017 | ☆ | ☆ |  | ☆ |  | ☆ |  | ☆ | ☆ | ☆ | 7 |
| He TD | 2017 | ☆ | ☆ |  | ☆ |  | ☆ |  | ☆ | ☆ |  | 6 |
| Hu PH | 2017 | ☆ | ☆ |  | ☆ |  | ☆☆ |  |  |  | ☆ | 8 |
| Lin YX | 2017 | ☆ | ☆ |  | ☆ |  | ☆☆ |  | ☆ | ☆ | ☆ | 8 |
| Li LL | 2018 | ☆ | ☆ |  | ☆ |  | ☆☆ |  | ☆ | ☆ |  | 8 |
| Yang ML | 2018 | ☆ | ☆ |  | ☆ |  | ☆☆ |  | ☆ | ☆ | ☆ | 8 |
| Yin ZH | 2018 | ☆ | ☆ |  | ☆ |  | ☆☆ |  | ☆ | ☆ | ☆ | 8 |
| Yuan ZY | 2018 | ☆ | ☆ | ☆ | ☆ |  | ☆ |  | ☆ | ☆ |  | 7 |
| Cui P | 2018 | ☆ | ☆ | ☆ | ☆ |  | ☆ |  | ☆ | ☆ |  | 7 |
| Abdollahzadeh | 2018 | ☆ | ☆ |  | ☆ |  | ☆☆ |  | ☆ | ☆ | ☆ | 8 |
| Hu C | 2019 | ☆ | ☆ |  | ☆ |  | ☆☆ |  | ☆ | ☆ |  | 7 |
| Li Z | 2019 | ☆ | ☆ |  | ☆ |  | ☆☆ |  | ☆ | ☆ | ☆ | 8 |
| Safari | 2019 | ☆ | ☆ |  | ☆ |  | ☆☆ |  | ☆ | ☆ |  | 7 |
| Wang GZ | 2019 | ☆ | ☆ |  | ☆ |  | ☆ |  | ☆ | ☆ |  | 6 |
| Wu | 2019 | ☆ | ☆ |  | ☆ |  | ☆☆ |  | ☆ | ☆ | ☆ | 8 |
| Wei MR | 2019 | ☆ | ☆ |  | ☆ |  | ☆☆ |  | ☆ | ☆ |  | 7 |
| Huang MC | 2019 | ☆ | ☆ | ☆ | ☆ |  | ☆☆ |  | ☆ | ☆ |  | 8 |
| Yang PJ | 2019 | ☆ | ☆ |  | ☆ |  | ☆☆ |  | ☆ | ☆ |  | 7 |
| Cao Q | 2020 | ☆ | ☆ |  | ☆ |  | ☆☆ |  | ☆ | ☆ | ☆ | 8 |
| Ghapanchi | 2020 | ☆ | ☆ | ☆ | ☆ |  | ☆☆ |  | ☆ | ☆ |  | 8 |
| Deng YJ | 2020 | ☆ | ☆ |  | ☆ |  | ☆☆ |  | ☆ | ☆ |  | 7 |
| Yu BQ | 2021 | ☆ | ☆ |  | ☆ |  | ☆☆ |  | ☆ | ☆ |  | 7 |
| Zhang HB | 2021 | ☆ | ☆ |  | ☆ |  | ☆ |  | ☆ | ☆ |  | 6 |
| Li WY | 2021 | ☆ | ☆ |  | ☆ |  | ☆☆ |  | ☆ | ☆ |  | 7 |
| Pei JS | 2021 | ☆ | ☆ | ☆ | ☆ |  | ☆☆ |  | ☆ | ☆ |  | 7 |
| Tan TB | 2021 | ☆ | ☆ | ☆ | ☆ |  | ☆☆ |  | ☆ | ☆ |  | 8 |
| Zhang JZ | 2021 | ☆ | ☆ |  | ☆ |  | ☆☆ |  | ☆ | ☆ | ☆ | 8 |
| Khalil | 2022 | ☆ | ☆ |  | ☆ |  | ☆ |  | ☆ | ☆ |  | 6 |
