# Supplementary material for: Six polymorphisms in the lncRNA H19 gene and the risk of cancer: a systematic review and meta-analysis
Source: BMC Cancer. 2023 Jul 21;23:688. doi: 10.1186/s12885-023-11164-y (PMC10362596; doi:10.1186/s12885-023-11164-y)
Supplement: Supplementary file 3 — Supplementary Material 3 [file 12885_2023_11164_MOESM3_ESM.doc]

**
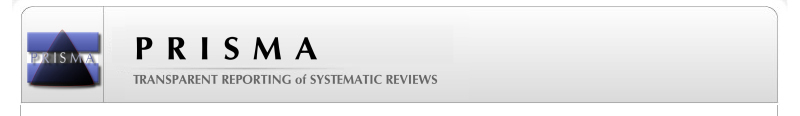
PRISMA 2009 Flow Diagram**

**Screening**

**Included**

**Eligibility**

**Identification**

Studies included in quantitative synthesis (meta-analysis)
(n = 40)

13 studies relevant to H19 rs2107425C/T

30 studies relevant to H19 rs217727C/T

26 studies relevant to H19 rs2839698G/A

10 studies relevant to H19 rs3741219T/C

12 studies relevant to H19 rs3924270C/G

4 studies relevant to H19 rs3741216A/T

Records identified through database searching (n = 471):

PubMed (n = 229), Embase (n = 76),

and Web of science (n = 166)

Additional records identified through other sources
(n = 0)

Records after duplicates removed
(n = 319)

Records screened
(n = 319)

Records excluded

(n = 191)

Full-text articles assessed for eligibility
(n = 128)

Excluded (n = 88)

Other diseases (n = 21)

Not relevant to gene (n = 72)

Without sufficient data (n = 9)

Inconsistent with HWE (n = 2)

Studies included in qualitative synthesis
(n = 40)
